# A regulatory network controlling ovarian granulosa cell death

**DOI:** 10.1038/s41420-023-01346-9

**Published:** 2023-02-20

**Authors:** Liu Yang, Xing Du, Siqi Wang, Chenggang Lin, Qiqi Li, Qifa Li

**Affiliations:** College of Animal Science and Technology, Nanjing Agricultural University, Nanjing, 210095 China

**Keywords:** Apoptosis, Gene expression

## Abstract

Follicular atresia triggered by granulosa cell (GC) apoptosis severely reduces female fertility and accelerates reproductive aging. GC apoptosis is a complex process regulated by multiple factors, regulatory axes, and signaling pathways. Here, we report a novel, small regulatory network involved in GC apoptosis and follicular atresia. miR-187, a miRNA down-regulated during follicular atresia in sows, maintains TGFBR2 mRNA stability in sow GCs by directly binding to its 5’-UTR. miR-187 activates the transforming growth factor-β (TGF-β) signaling pathway and suppresses GC apoptosis via TGFBR2 activation. NORHA, a pro-apoptotic lncRNA expressed in sow GCs, inhibits TGFBR2-mediated activation of the TGF-β signaling pathway by sponging miR-187. In contrast, NORFA, a functional lncRNA associated with sow follicular atresia and GC apoptosis, enhances miR-187 and TGFBR2 expression by inhibiting NORHA and activating NFIX. Our findings define a simple regulatory network that controls GC apoptosis and follicular atresia, providing new insights into the mechanisms of GC apoptosis, follicular atresia, and female fertility.

## Introduction

Transforming growth factor-β (TGF-β) signaling plays essential roles in almost all key events during gametogenesis and embryonic development, and in maintaining adult tissue homeostasis and normal physiological functions in mammals [1, 2]. Dysfunction or dysregulation of TGF-β signaling can result in lethality, embryo-fetal malformations, and multiple diseases [3, 4]. Canonical TGF-β signaling is initiated by the binding of extracellular ligands to cell surface receptors, leading to SMAD2/3 activation in the cytoplasm, which forms a complex with SMAD4. The newly formed cytoplasmic complex then translocates to the nucleus, where it implements signal transduction [2]. TGFBR2 is the primary ligand binding receptor, playing an integral role in the extracellular TGF-β signal transmission to the cytoplasm. Compelling evidence suggests that TGFBR2 is involved in a broad range of cellular processes including proliferation [5], differentiation [6], death [7], stemness [8], invasion [9], and infiltration [10].

Given the central role of TGFBR2 in TGF-β signal transduction, which strongly impacts development and health, pharmacological targeting of TGFBR2 to modulate TGF-β signaling offers potential clinical and production applications [11, 12]. TGF-β1 is the most important TGFBR2 activating ligand. Once bound to TGF-β1, the intracellular domain of TGFBR2 exhibits constitutive serine/threonine kinase activity [13]. In addition to TGF-β1, several modifying factors, including casitas B-lineage lymphoma (Cbl) [14], ubiquitin-specific peptidase 9 X-linked (USP9X) [15], asparagine-linked glycosylation 10 (ALG10) [8], and programmed death-ligand 1 (PD-L1) [16], also directly activate or maintain levels of activated TGFBR2 protein through neddylation, ubiquitination, glycosylation modifications, and lysosomal degradation. TGFBR2 promoter binding, transcription factors including SMAD4 [17] and GABPA [9] activate TGFBR2 transcription, whereas MYOCD [18] inhibits TGFBR2 transcription. At the post-transcriptional level, several miRNAs have been reported to directly interact with the 3′ untranslated region (UTR) of their mRNAs to decrease TGFBR2 levels [19, 20]. However, TGFBR2 regulation via 5′-UTR targeting has not been clearly demonstrated.

In sow granulosa cells (GCs), we previously demonstrated that TGFBR2 is a vital anti-apoptotic factor that is downregulated by miRNAs that bind to its 3′-UTR [12, 15]. TGFBR2 can also be induced by binding SMAD4 to its promoter [17], and its protein levels are maintained by the deubiquitinase activity of USP9X [15] in sow GCs. In this study, using the 5′-UTR as the target region, we investigated the regulatory mechanism of TGFBR2 expression in sow GCs. We showed that miR-187, which regulates sow follicular atresia, directly binds to the 5′-UTR of TGFBR2 mRNA to maintain its stability. miR-187 functions in coordination with competing for endogenous RNA (ceRNA) NORHA (a pro-apoptotic lncRNA in sow GCs that is strongly involved in sow follicular atresia [21]) and NORFA. NORFA is a modulator of NORHA transcription and the first lncRNA shown to promote sow fertility traits by inhibiting GC apoptosis and follicular atresia [22], and by regulating TGF-β signaling through TGFBR2. We have constructed a simple molecular network regulating GC apoptosis and follicular atresia that involves TGFBR2, NFIX, miR-187, NORHA, and NORFA as hub genes.

## Results

### miR-187 directly targets the 5′-UTR of sow TGFBR2 gene

**Fig. 1:**
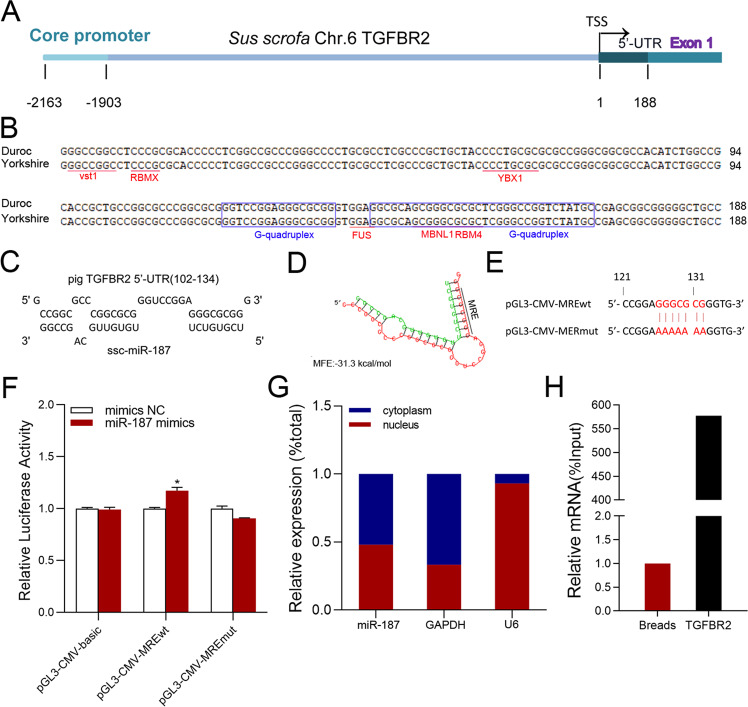
miR-187 targets the 5′-UTR of the sow TGFBR2 gene. **A** Schematic diagram of TGFBR2 5′ regulatory region. **B** Sequence alignment of TGFBR2 5′-UTR from Duroc and Yorkshire pigs. RBP-binding elements are indicated by underlining. RNA G-quadruplexes are indicated by blue boxes. **C** The MRE motif of miR-187 in TGFBR2 5′-UTR. **D** Prediction of minimum free energy (MFE) of binding miR-187 and TGFBR2 5’-UTR using RNAhybrid. **E** Schematic showing the reporter vectors of TGFBR2 5’-UTR containing wild-type and mutated MRE of miR-187. **F** Luciferase reporter assays. **G** Subcellular distribution of miR-187 in sow GCs. Nuclear and cytoplasmic RNAs were isolated from GCs, and levels of miR-187 and internal reference genes (U6 in the nucleus and GAPDH in the cytoplasm) were detected using qPCR. **H** RNA pull-down assay. TGFBR2 5′-UTR were pulled down using biotin-labeled probes of miR-187 in GCs and detected by qPCR. Data represent means ± SEM for three independent experiments. *p < 0.05.

According to our previous 5′RACE assay [17], the full-length Yorkshire sows TGFBR2 5′-UTR is 188 bp, which is completely consistent with Duroc pigs (Fig. 1A, B). Several classic *cis*-regulatory elements have been detected in the sow TGFBR2 5′-UTR, including RNA binding protein (RBP) binding elements such as RBMX and YBX1 (Fig. 1B). Putative miRNA recognition element (MRE) motifs of 24 miRNAs have been detected in this region (Table S8). In addition, two potential RNA G-quadruplexes (four-stranded structures involved in translation, chromatin structure, and RNA modifications [23]) are predicted at 118–133 nt and 139–163 nt in the TGFBR2 5′-UTR.

Interestingly, among the putative miRNAs that target the TGFBR2 5′-UTR, only miR-187, like TGFBR2, is downregulated during sow follicular atresia [21, 24]. A potential MRE motif for miR-187 was discovered at 102–134 nt in the sow TGFBR2 5′-UTR, with a maximal potential binding ability (minimum free energy (MFE) of −31.3 kcal/mol) (Fig. 1C, D). In vertebrates, miR-187 mature sequences were completely consistent (Fig. S1). To investigate the effect of miR-187 on TGFBR2 5′-UTR activity, reporter vectors for TGFBR2 5′-UTR were constructed (Fig. 1E). Luciferase reporter assays revealed that miR-187 markedly induced the activity of the reporter vector of TGFBR2 5′-UTR, but did not affect the activity of the reporter vector of TGFBR2 5′-UTR with the mutated MRE motif of miR-187 (Fig. 1F), revealing that miR-187 induces TGFBR2 5′-UTR activity via the MRE motif of miR-187. Furthermore, a subcellular localization assay showed that the abundance of miR-187 was higher in GC cytoplasm than in the nucleus (Fig. 1G). An RNA pull-down assay showed that miR-187 directly binds to the MRE motif of miR-187 in the 5′-UTR (Fig. 1H). Taken together, these data indicate that miR-187 directly targets the 5′-UTR in the cytoplasm to induce TGFBR2 5′-UTR activity in sow GCs.

### miR-187 maintains TGFBR2 levels in GCs by enhancing TGFBR2 mRNA stability

**Fig. 2:**
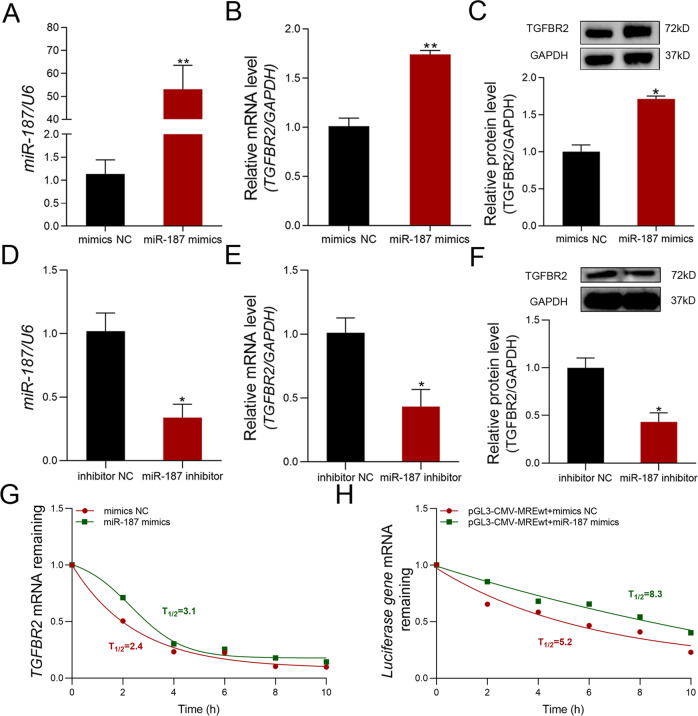
miR-187 maintains TGFBR2 levels in GCs by influencing their stability. **A**–**C** After incubating GCs with miR-187 mimics, levels of miR-187 (**A**), TGFRB2 mRNA (**B**), and TGFBR2 protein (**C**) were measured. **D**–**F** After incubating GCs with miR-187 inhibitor, levels of miR-187 (**D**), TGFRB2 mRNA (**E**), and TGFBR2 protein (**F**) were measured. **G**, **H** mRNA stability assays. **G** After transfection of miR-187 mimics or mimics NC into GCs for 10 h, ActD was added, then TGFBR2 mRNA levels were detected at 0, 2, 4, 6, 8, and 10 h. **H** After co-transfection with the plasmid pGL3-CMV-MREwt and miR-187 mimics or mimics NC into KGN cells for 10 h, ActD was added, then firefly luciferase gene mRNA levels were detected at 0, 2, 4, 6, 8, and 10 h. Data are represented as means ± SEM for three independent experiments. *p < 0.05; **p < 0.01.

To investigate whether miR-187 influences TGFBR2 levels, sow GCs were treated with miR-187 oligonucleotides. TGFBR2 mRNA and protein levels increased in GCs treated with miR-187 mimics (Fig. 2A–C and Fig. S2A). In contrast, TGFBR2 mRNA and protein levels were downregulated in GCs treated with the miR-187 inhibitor (Fig. 2D–F and Fig. S2B). Taken together, these data suggest that miR-187 strongly influences TGFBR2 levels in sow GCs.

*Trans*-acting factors (e.g., RBPs) targeting the 5′-UTR have been demonstrated to maintain transcript levels by affecting mRNA stability [25]. Therefore, we examined whether miR-187 influences TGFBR2 mRNA stability in GCs. To this end, GCs were treated with miR-187 mimics and subsequently exposed to ActD, a transcriptional inhibitor. TGFBR2 mRNA levels in GCs treated with miR-187 mimics were significantly higher than those in the control group at 2, 4, 6, 8, and 10 h (Fig. S3A), and the respective mRNA half-lives were 3.1 h and 2.4 h (Fig. 2G), indicating that miR-187 enhances TGFBR2 mRNA stability in sow GCs. We also measured firefly luciferase gene mRNA levels in KGN cells in which transcription was blocked with ActD at 2, 4, 6, 8, and 10 h after co-transfection with the reporter vector of TGFBR2 5′-UTR and miR-187 mimics. As expected, compared to the control group (half-life was 5.2 h), significantly enhanced luciferase mRNA stability was observed in miR-187-overexpressing cells (half-life was 8.3 h) (Fig. 2H and S3B), in contrast with the in vitro results of transcription activity (Fig. 1F) and transcription levels (Fig. 2B, E). Together, these data reveal that miR-187 maintains TGFBR2 levels in sow GCs by enhancing TGFBR2 mRNA stability.

### miR-187 represses GC apoptosis by activating TGF-β signaling

**Fig. 3:**
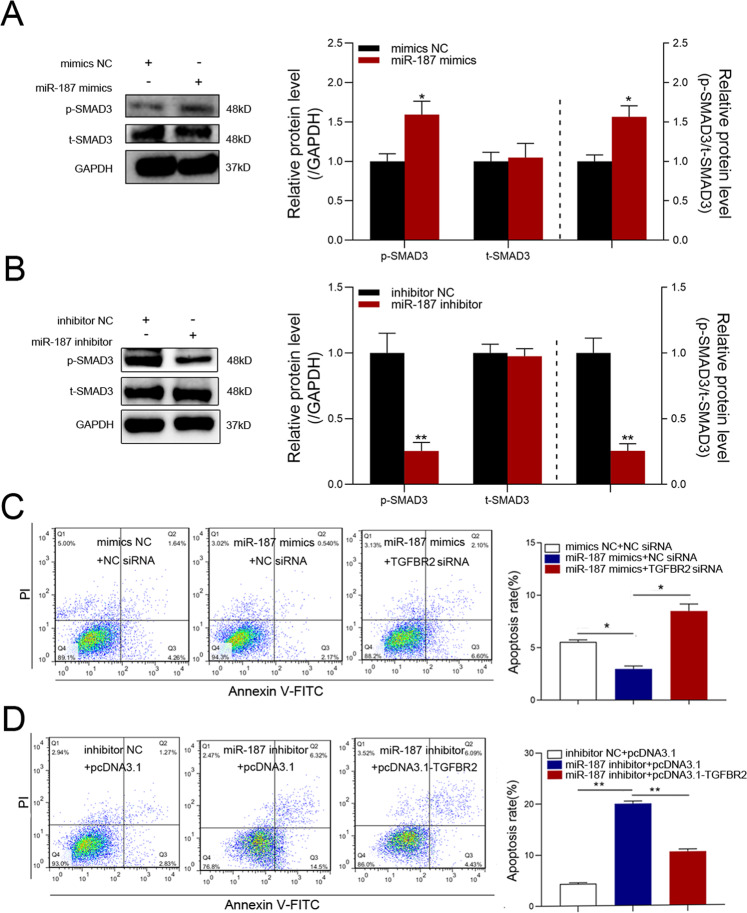
miR-187 regulates TGF-β signaling and GC apoptosis by targeting TGFBR2. **A**, **B** miR-187 activates TGF-β signaling in GCs. GCs were transfected with miR-187 mimics (**A**) or inhibitor (**B**), and phosphorylated SMAD3 (p-SMAD3) and total SMAD3 (t-SMAD3) protein levels were detected using western blot. **C**, **D** FACS detection of apoptosis rate in GCs after co-treatment of miR-187 mimics and TGFBR2-siRNA (**C**) or miR-187 inhibitor and pcDNA3.1-TGFBR2 (**D**). Data are represented as means ± SEM for three independent experiments. *p < 0.05; **p < 0.01.

To test whether miR-187 activates TGF-β signaling in GCs, we measured phosphorylated SMAD3 (p-SMAD3) levels in GCs treated with miR-187 oligonucleotides. As expected, significantly elevated p-SMAD3 levels were observed in miR-187-overexpressing GCs, whereas total SMAD3 protein levels did not change significantly (Fig. 3A and Fig. S2C). In contrast, p-SMAD3 levels were significantly decreased in miR-187-silenced GCs (Fig. 3B and Fig. S2D). This data suggests that miR-187 is an activator of TGF-β signaling in GCs.

We predicted 560 putative targets of miR-187 using online tools (Table S9) and found that these targets were significantly enriched in apoptosis-related pathways and cell functions by Kyoto Encyclopedia of Genes and Genomes (KEGG) analysis (Fig. S4). Next, we analyzed the role of miR-187 in sow GC apoptosis. A significantly reduced apoptosis rate was observed in miR-187-overexpressing GCs, whereas a significantly enhanced apoptosis rate was seen in miR-187-silenced GCs (Fig. 3C), indicating that miR-187 inhibits GC apoptosis. To confirm whether miR-187 reduces GC apoptosis via its target, TGFBR2, two co-transfection experiments were performed. As expected, silencing of TGFBR2 suppressed miR-187-induced downregulation of the apoptosis rate, whereas TGFBR2 overexpression suppressed miR-187-inhibitor-induced upregulation of the apoptosis rate in GCs (Fig. 3D). Taken together, our results suggest that miR-187 suppresses apoptosis in GCs by enhancing TGFBR2 activation.

### NORHA suppresses TGFBR2 expression by sponging miR-187

**Fig. 4:**
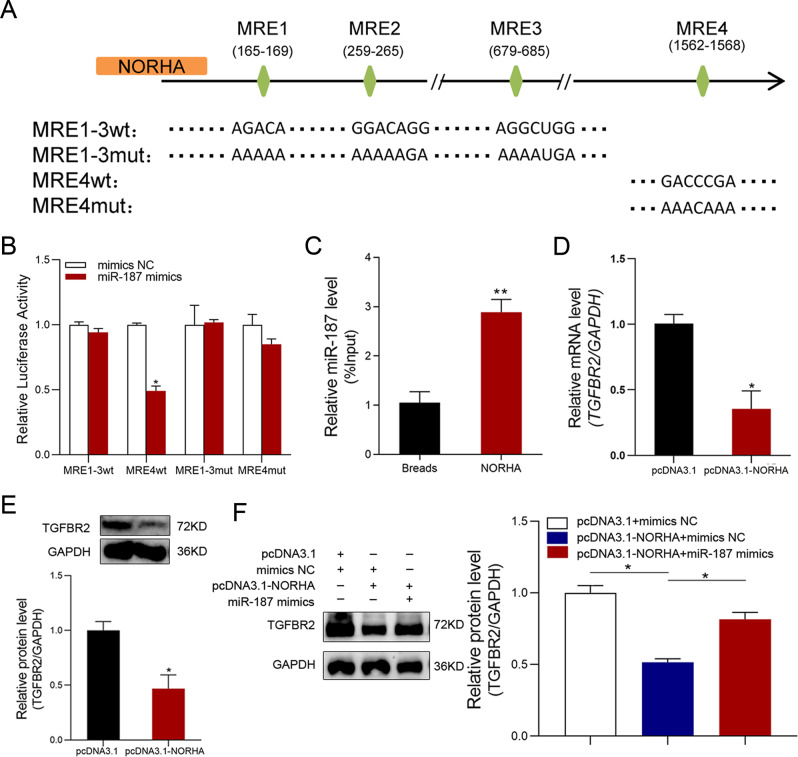
NORHA regulates TGF-β signaling by adsorbing miR-187. **A** Schematic of reporter vectors expressing NORHA containing wild-type or mutated MREs of miR-187. **B** Luciferase reactivity was determined and normalized to the NC group. **C** RNA pull-down assay. miR-187 was pulled down using biotin-labeled probes for NORHA containing MRE4 motif in GCs and then detected using qPCR. **D**, **E** GCs were treated with plasmid pcDNA3.1-NORHA, TGFBR2 mRNA levels were detected using qPCR (**D**), and protein levels were detected using western blotting (**E**). **F** TGFBR2 protein levels in GCs after co-treatment of pcDNA3.1-NORHA and miR-187 mimics were detected by western blot. Data are represented as means ± SEM for three independent experiments. *p < 0.05; **p < 0.01.

Both miR-187 and lncRNA NORHA are cytoplasmic ncRNAs expressed in sow GCs [21]. Four putative MRE motifs of miR-187 were detected at 165–169 nt (MRE1), 259–265 nt (MRE2), 679–685 nt (MRE3), and 1562–1568 nt (MRE4) of NORHA using an RNAhybrid tool (Fig. S5). Therefore, we speculate that NORHA is a potential ceRNA of miR-187. To assess this, reporter vectors of NORHA, containing wild-type and mutated MRE motifs for miR-187, were generated (Fig. 4A). Luciferase reporter assays showed that miR-187 exerted an inhibitory effect on the luciferase activity of the NORHA reporter vector containing the wild-type MRE4 motif, whereas the NORHA reporter vector containing wild-type or mutated MRE1–3 motifs, or a mutated MRE4 motif (Fig. 4B) was not affected, indicating that miR-187 interacts with the MRE4 motif of NORHA. In addition, an RNA pull-down assay using a biotin-labeled probe for NORHA containing the MRE4 motif showed that NORHA directly binds to miR-187 in sow GCs (Fig. 4C). Combining the above results, we concluded that NORHA sponges miR-187 in GCs via its MRE4 motif.

We sought to determine whether NORHA sponging of miR-187 affects the expression of the latter target TGFBR2 in sow GCs. We found that TGFBR2 levels were markedly decreased by NORHA overexpression (Fig. 4D, E and Fig. S2E). Co-transfection experiments revealed that miR-187 reversed NORHA-induced downregulation of TGFBR2 levels in GCs (Fig. 4F and Fig. S2F). Taken together, our data suggest that NORHA suppresses TGFBR2 expression in sow GCs by sponging miR-187.

### NORHA inactivates TGF-β signaling and promotes GC apoptosis through a miR-187/TGFBR2 axis

**Fig. 5:**
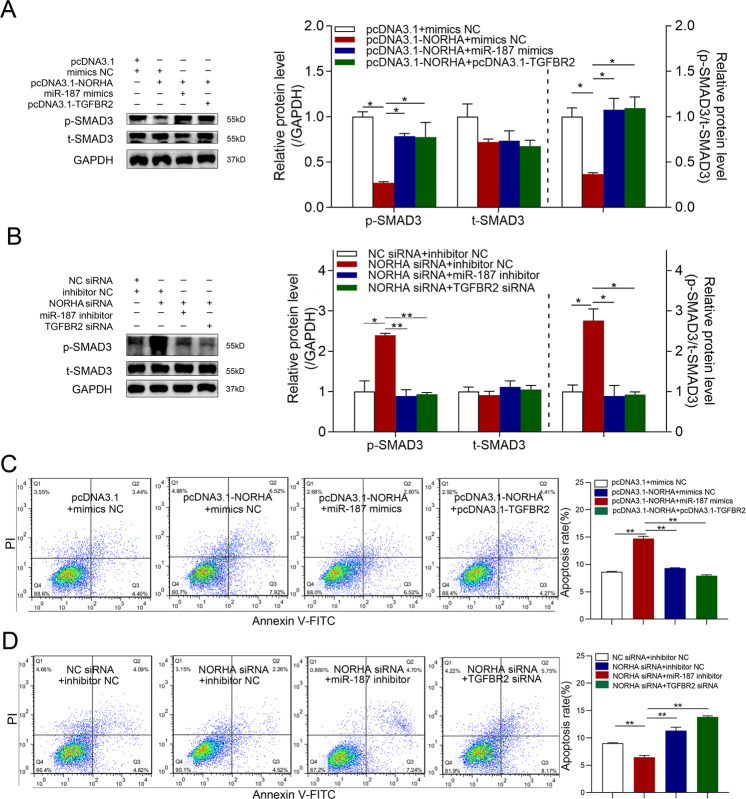
NORHA regulates TGF-β signaling and GC apoptosis by a miR-187/TGFBR2 axis. **A**, **B** Western blotting was used to detect p-SMAD3 and t-SMAD3 protein levels after co-transfection with pcDNA3.1-NORHA and miR-187 mimics or pcDNA3.1-NORHA and pcDNA-TGFBR2 (**A**), or NORHA-siRNA and miR-187 inhibitor or NORHA-siRNA and TGFBR2-siRNA (**B**). **C**, **D** FACS analysis was used to detect the apoptosis rate of GCs after co-treatment with pcDNA3.1-NORHA and miR-187 mimics or pc3.1DNA-NORHA and pcDNA3.1-TGFBR2 (**C**), or NORHA-siRNA and miR-187 inhibitor or NORHA-siRNA and TGFBR2-siRNA (**D**). Data are represented as mean ± SEM for three independent experiments. *p < 0.05; **p < 0.01.

We next investigated the regulatory effect of NORHA on TGF-β signaling activity in GCs. p-SMAD3 levels were significantly decreased in NORHA-overexpressing GCs, whereas they were significantly elevated in NORHA-silenced GCs (Fig. 5A, B), indicating that NORHA inactivates TGF-β signaling in GCs. Furthermore, co-transfection experiments revealed that both miR-187 and TGFBR2 reversed NORHA-induced downregulation of p-SMAD3 levels in GCs (Fig. 5A, B and Fig. S2G, H). Our results indicate that NORHA represses TGF-β signaling by regulating the miR-187 and TGFBR2 axis in GCs.

In our previous study, NORHA promoted apoptosis in sow GCs [21]. These results showed that miR-187 represses GC apoptosis by promoting TGFBR2 expression. Therefore, we investigated whether NORHA affects GC apoptosis by adsorbing miR-187. Fluorescence-activated cell sorting (FACS) analysis revealed that the addition of miR-187 mimics or TGFBR2 overexpression plasmids reversed the increase in apoptosis induced by NORHA (Fig. 5C, D). The data shows that NORHA promotes apoptosis in GCs by regulating the miR-187 and TGFBR2 axis and that NORHA/miR-187/TGFBR2 is a novel signaling pathway for regulating GC apoptosis.

### A small regulatory network modulates GC apoptosis and follicular atresia

**Fig. 6:**
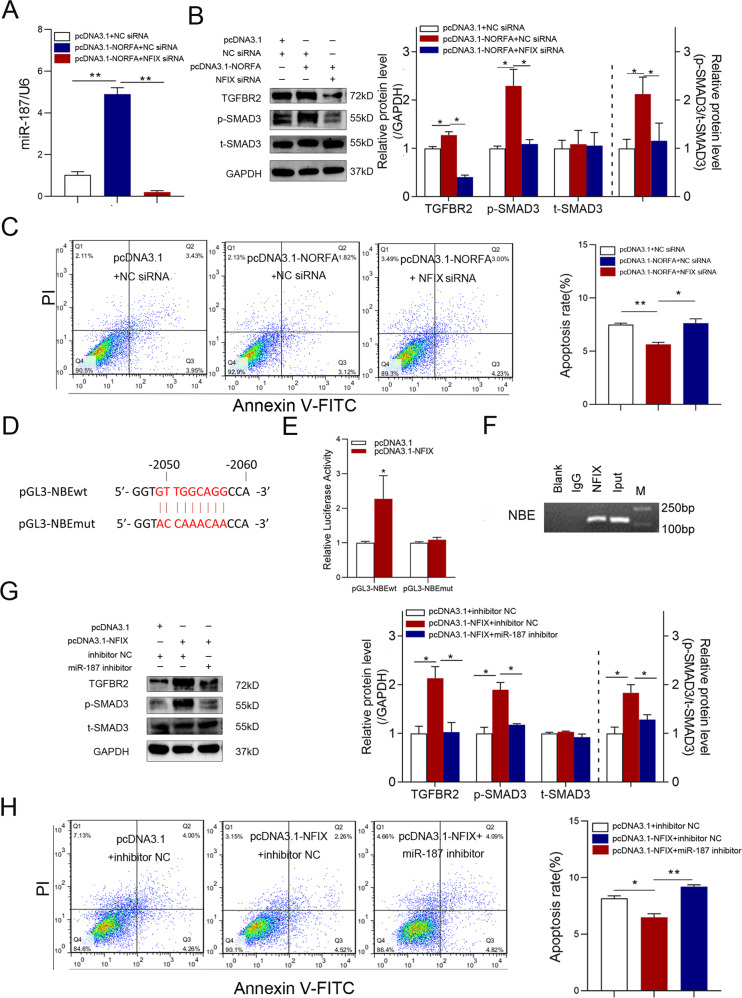
NORFA regulates TGF-β signaling and apoptosis in sow GCs through an NFIX/miR-187 axis. **A** qPCR detection of miR-187 levels after co-transfection with pcDNA3.1-NORFA and NFIX-siRNA. **B** Western blot detection of TGFBR2, p-SMAD3, and t-SMAD3 protein levels after co-transfection with pcDNA3.1-NORFA and NFIX-siRNA. **C** FACS detection of apoptosis rate after co-treatment with pcDNA3.1-NORFA and NFIX-siRNA. **D** Schematic representing the reporter vectors for the miR-187 promoter containing wild-type and mutated NBE motifs. **E** Luciferase activity was determined in GCs co-transfection with plasmid pcDNA3.1-NFIX and pGL3-NBEwt or pGL3-NBEmt. **F** ChIP detection of NFIX bound to the miR-187 promoter using an NFIX-specific antibody. **G** Western blot detection of TGFBR2, p-SMAD3, and t-SMAD3 protein levels after co-transfection with pcDNA3.1-NFIX and miR-187 inhibitor. **H** FACS analysis detection of apoptosis rate after co-treatment with pcDNA3.1-NFIX and miR-187 inhibitor. Data represent means ± SEM for three independent experiments. *p < 0.05; **p < 0.01.

Our previous studies showed that miR-187 is downregulated in GCs by knockdown of NORFA, a functional lncRNA regulating sow GC apoptosis and follicular atresia, and regulated by the NORFA-induced transcription factor NFIX [22, 24]. Here, we show that NORFA-induced upregulation of miR-187 and TGFBR2 levels and activation of TGF-β signaling was also repressed by silencing the transcription factor NFIX (Fig. 6A, B and Fig. S6A). In addition, NFIX silencing rescues the inhibition of GC apoptosis caused by NORFA overexpression (Fig. 6C), indicating that NORFA controls the miR-187 expression and its functions in sow GCs via NFIX.

Interestingly, an NFIX-binding element (NBE) motif was observed in the miR-187 promoter region (Fig. 6D). Luciferase reporter assays showed that NFIX significantly induced miR-187 promoter activity through the NBE motif (Fig. 6E). A Chromatin immunoprecipitation (ChIP) assay verified that NFIX binds directly to the NBE motif in the miR-187 promoter (Fig. 6F). Co-transfection experiments showed that NFIX induced TGFBR2 expression and activated TGF-β signaling in GCs, which was reversed by miR-187 knockdown (Fig. 6G and Fig. S6A). Furthermore, miR-187 knockdown reversed the NFIX-induced decrease in the GC apoptosis rate (Fig. 6H). Together, these results suggest that the NORFA-NFIX loop regulates TGF-β signaling and cell apoptosis by inducing miR-187 expression in sow GCs.

**Fig. 7:**
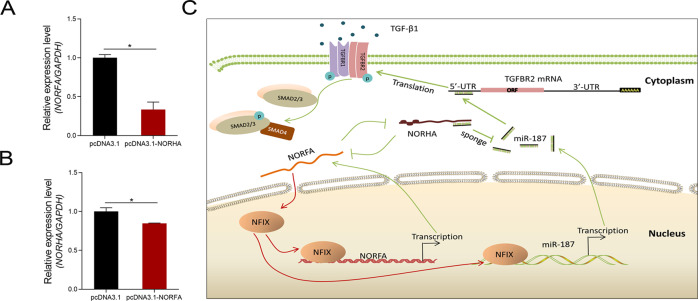
A small regulatory network of functional genes for GC apoptosis and follicular atresia. **A** NORFA levels were measured using qPCR in NORHA-overexpressing GCs. **B** NORHA levels were measured using qPCR in NORFA-overexpressing GCs. **C** This network comprises three ncRNAs (miR-187, NORHA, and NORFA), two feedback loops (NORFA-NFIX and NORHA-NORFA), and one signaling pathway (TGF-β). Four hub genes (NORHA, NORFA, miR-187, and TGFBR2) in this network are strongly associated with follicular atresia by influencing GC apoptosis. Red dotted arrows have been demonstrated by our previous studies [22, 24]. Data represent means ± SEM for three independent experiments. *p < 0.05.

In addition, NORHA suppresses NORFA levels in sow GCs (Fig. 7A). Similarly, NORFA suppresses NORHA expression in GCs (Fig. 7B). Combined with our previous study [25], the above results roughly outline a sow GC apoptosis and follicular atresia-related small regulatory network composed of three ncRNAs (miR-187, NORHA, and NORFA), two feedback loops (NORFA-NFIX and NORHA-NORFA), and one signaling pathway (TGF-β) (Fig. 7C).

## Discussion

Sow fertility exerts a significant economic impact on the swine industry as it directly relates to production efficiency. Follicular development and ovulation are considered the most important initial factors determining sow reproductive potential, whereas follicular atresia and degeneration triggered by GC apoptosis are limiting factors impacting sow fertility. Follicular atresia and GC apoptosis are complex processes governed by multiple factors. Therefore, it is more important to determine the regulatory network that controls follicular atresia or GC apoptosis than to investigate the regulatory effects of a single factor on follicular atresia or GC apoptosis. In the present study, we identified a small regulatory network that controls GC apoptosis via TGFBR2, NFIX, miR-187, NORHA, and NORFA. Our findings provide a new feasible scheme for analyzing the mechanism of follicular atresia and GC apoptosis by building a regulatory network composed of multiple key factors.

Transcriptional control in 5′-terminal promoters and enhancers, and post-transcriptional control mechanisms in 3′-UTRs have long been the focus of gene expression regulation [26, 27]. The former is mainly bound and controlled by transcription factors and chromatin remodelers, while the latter is mainly bound and controlled by RBPs and miRNAs [28, 29], through direct binding to specific promoter regions, enhancers, or 3′-UTRs. However, 5′-UTRs have received little attention, although some binding elements of RBPs have been identified through multi-omics technologies [30]. In this study, we identified multiple *cis*-acting elements in the TGFBR2 5′-UTR and showed that miR-187 maintains its mRNA stability in sow GCs through direct interaction with the 5′-UTR. Consistent with this, miR-23a/b-3p induces mRNA stability of Srebp-1c and Fas in hepatocytes by binding to the 5′-UTR [31]. Furthermore, miR-1254 [25], miR-6981 [32], and miR-760 [33] bind directly to their target mRNA 5′-UTRs. Additionally, miR-122 induces genome stability and stimulates viral translation via interaction with a viral 5′-UTR [34]. A recent study showed that miR-182-5p binds to the 5′-UTR to decrease Nr1d1 mRNA levels in mouse adipose tissues [35]. Together, our findings not only fill the gap in understanding TGFBR2 5′-UTR regulation but also provide new evidence for miRNA-mediated post-transcriptional control via 5′-UTR binding. Several studies have also demonstrated that RBPs and G-quadruplex-binding proteins such as DHX36 control mRNA decay and translation by interacting with 5′-UTRs [36, 37]. Subsequently, we will continue to investigate the role and mechanism of other predicted cis-acting elements in the TGFBR2 5′-UTR.

We showed that miR-187 functions as an anti-apoptotic factor in sow GCs by activating TGFBR2-mediated TGF-β signaling. In the ovary, miR-187 is strongly involved in sow follicular atresia [21]. Follicular atresia is known to be triggered by GC apoptosis; thus, our recent study further demonstrates that miR-187 is an inhibitor of sow follicular atresia. In another female reproductive organ, the uterus, miR-187 targets integrin beta8 (ITGB8), a subunit of integrin beta, to inhibit proliferation and focal adhesion kinase (FAK) activity in goat endometrial epithelial cells [38]. In addition to cells in female reproductive organs, miR-187 is a multifunctional epigenetic regulator in various cell types [39–42]. In human trophoblast cells, miR-187 induces apoptosis and represses proliferation, migration, and invasion by targeting BCL6-mediated activation of the PI3K/AKT signaling [41]. In cells involved in various pathologies, miR-187 also plays essential roles in various cellular processes, including restraining proliferation, promoting cell cycle progression, accelerating apoptosis, and decelerating the migration and invasion of pituitary adenoma cells by elevating TESC and inhibiting NF-κB signaling [42]. Our findings define a new function of miR-187, which is to provide a potential small-molecule regulator for maintaining active TGF-β signaling, rescuing follicular atresia, and prolonging female fertility. Further work is needed to clarify its role in female reproduction in vivo.

The human miR-187 precursor (pre-miR-187) is transcribed from an intergenic region on human chromosome 18. In human monocytes, lipopolysaccharide (LPS) and its stimulated IL-10 synergistically induce the recruitment of RNA polymerase II, an enzyme that catalyzes DNA transcription to synthesize precursor RNA in the genomic region encoding pre-miR-187, suggesting that LPS and IL-10 control miR-187 expression at the transcriptional level [39]. Like human miR-187, pig miR-187 is also an intergenic miRNA located between C6H18orf21 and GALNT1 genes on pig chromosome 6. Here, we showed that NFIX activates miR-187 transcription by directly binding to the promoter of sow pre-miR-187, mediates NORFA induction of miR-187, activates the TGF-β signaling pathway, and inhibits GC apoptosis. NFIX is an important member of the NFI transcription factor family, which functions in GC apoptosis by transcriptionally activating and repressing female reproduction-related miRNAs (e.g., miR-27a and miR-126) and lncRNAs (e.g., NORFA) [22, 24, 43]. In this study, we also showed that the regulation of TGFBR2 by cytoplasmic miR-187 is competitively inhibited by the pro-apoptotic lncRNA NORHA in sow GCs [21]. Interestingly, in addition to competitive binding by lncRNAs, miR-187 is also competitively bound by circRNAs (e.g., hsa_circRNA_104348) and mRNAs (e.g., CXCR5) [40, 44]. Together, our findings not only reveal a new mechanism underlying the regulation of miR-187 expression and function but also define a small regulatory network for regulating GC apoptosis in sow GCs, with miR-187 as the core molecule.

Our results and those of previous studies demonstrate that protein-coding genes, including TGFBR2 and NFIX [12, 22] and miR-187 (this study), and lncRNAs, including NORHA [20] and NORFA [22], strongly regulate follicular atresia by influencing GC apoptosis. In this study, we further revealed a regulatory relationship between these factors in sow GCs and constructed a small regulatory network for controlling GC apoptosis and follicular atresia, with these factors as hub genes. This is different from previous studies on the regulatory mechanism of sow follicular atresia and GC apoptosis, which mainly focused on a single factor [45, 46], regulatory axis [47], or signaling pathway [48, 49]. Our findings define, for the first time, a small regulatory network that affects follicular atresia and GC apoptosis in sows, providing a new perspective for revealing the mechanism of complex processes such as GC apoptosis and follicular atresia. Interestingly, we also noted that the core members of this small regulatory network, including TGFBR2, NFIX, miR-187, NORHA, and NORFA are all located on the quantitative trait locus (QTL) for sow fertility traits (https://www.animalgenome.org/cgi-bin/QTLdb/SS/index). In the future, we will identify variants in these core members and investigate the association between these variants and sow fertility traits to understand how broadly this regulatory network controls sow fertility traits.

In conclusion, we have demonstrated support for a novel model involving the post-transcriptional control of TGFBR2 in sow GCs, that is, miR-187 maintains TGFBR2 mRNA stability through direct interaction with its 5′-UTR. Functionally, miR-187 inhibits GC apoptosis by activating TGFBR2-mediated TGF-β signaling. Interestingly, the NORFA-inducing transcription factor NFIX and downregulation of NORHA have been identified as direct modulators of miR-187, together forming a regulatory network that controls GC apoptosis. Our findings identify multiple potential small molecules or non-steroidal regulators for rescuing follicular atresia and potentially prolonging female fertility and provide a new perspective for revealing mechanisms of complex processes such as GC apoptosis and follicular atresia.

## Materials and methods

### Motif analysis

RBP binding elements in the sow TGFBR2 5’-UTR were predicted using bioinformatics (http://www.bioinformatics.com.cn). MREs in TGFBR2 5’-UTR were predicted using the online tools miRBase (https://www.mirbase.org/) and RNAhybrid (https://bibiserv.cebitec.unibielefeld.de/rnahybrid/). RNA G-quadruplexes were predicted using pqsfinder (https://pqsfinder.fi.muni.cz/). TargetScan (http://www.targetscan.org/vert_80/), starBase (http://starbase.info/), miRTarBase (https://maayanlab.cloud/ Harmonizome/resource/MiRTarBase), and mirdb (http://mirdb.org/) were used to predict targets containing the MRE motif of miR-187, and Kobas (http://kobas.cbi.pku.cn/) was used to analyze the KEGG.

### Plasmid construction

Overexpression constructs pcDNA3.1-TGFBR2, pcDNA3.1-NORHA, pcDNA3.1-NORFA, and pcDNA3.1-NFIX were previously generated by our group [15, 21, 22]. The CMV promoter was isolated from the pcDNA3.1 expression vector and inserted between the *Kpn*I and *Sac*I restriction sites of the pGL3-basic vector (Promega, Madison, WI) to construct the pGL3-CMV vector. TGFBR2 5′-UTRs containing wild-type or mutated miR-187 MRE were synthesized by TsingKe (Beijing, China) and cloned into the pGL3-CMV vector to construct the TGFBR2 5′-UTR reporter vector pGL3-CMV-MREwt or pGL3-CMV-MREmut. Reporter vectors of a miR-187 promoter containing an NBE or NORHA containing miR-187 MRE were also synthesized by TsingKe (Beijing, China). Primers used are listed in Supplementary Table S1.

### Cell preparation and transient transfection

The human GC line KGN was purchased from Shanghai GuanDao Biological Engineering Co., Ltd., and has been recently validated by STR and tested for mycoplasma contamination. KGN was cultured in 12-well plates with Dulbecco’s Modified Eagle Medium (DMEM) (#SH30022.01, HyClone, Wuhan, China) containing 10 % fetal bovine serum (FBS) (#10099, Gibco, Grand Island, NY) in a 37 °C incubator containing 5% CO_2_ to reach appropriate confluency for transfection. GCs used in the experiments were obtained from 150 sexually mature commercial sows from Zhushun Biotech (Nanjing, China). Sow GCs were isolated from ovarian follicles 3–5 mm in diameter, and ovaries were collected from mature commercial sows. All GCs cells were mixed together and washed twice with phosphate-buffered saline (PBS) (#SH30256.01, HyClone, Wuhan, China) containing penicillin-streptomycin (PS) (#15140-122, Gibco, Grand Island, NY), then seeded into plates to ensure randomization of control and experimental groups and incubated for 24 h at 37 °C in a 5% CO_2_ incubator. We performed a randomized grouping to hide. Cells were washed and transfected with plasmids and oligonucleotides using Lipofectamine 3000 (#L3000015, Invitrogen, Carlsbad, CA, USA) in Opti-MEM (#31985070, Gibco, Grand Island, NY). Oligonucleotides were designed and synthesized by GenePharma (Shanghai, China) and are listed in Supplementary Table S2. All animal experiments were approved by the Animal Ethics Committee of Nanjing Agricultural University.

### Luciferase reporter assays

Cells and lysates were collected 48 h after treatment. Following the manufacturer’s instructions, a Duo-Lite Luciferase Assay System (#DD1205; Vazyme, Nanjing, China) was used to detect luciferase activity. Luciferase activity was calculated by dividing firefly luciferase activity by Renilla luciferase activity.

### RNA extraction and quantitative PCR (qPCR)

Extraction and reverse transcription of total RNA were carried out as described previously [15]. Transcription levels were determined using AceQ qPCR SYBR Green Master Mix (#Q111-02, Vazyme, Nanjing, China) in a QuantStudio™ 7 Flex Real-Time PCR System (Thermo Fisher Scientific, Waltham, MA, USA). Levels of miRNA and protein-coding genes were calculated using 2^−∆∆ct^ and normalized to housekeeping genes U6 and GAPDH, respectively. Primers used for qPCR are listed in Supplementary Table S3.

### Cytoplasmic and nuclear fractionation

When GCs exceeded 90% confluency in six-well plates, they were washed with PBS and digested with pre-warmed trypsin-EDTA (0.5 %). Supernatants were discarded after centrifugation at 1500×*g* for 5 min, and cell pellets were incubated in 500 μL PBS for 10 min on ice. Next, 0.1% Nonidet P-40 (#N8030, Solarbio, Beijing, China) was added and mixed well, then cell lysates were centrifuged at 12000×*g* for 5 min. Afterward, 500 μL TRIzol (#15596018, Invitrogen, Waltham, MA, USA) was added to each supernatant and pellet to extract total RNA. Reverse transcription reactions and qPCR were then performed as described above.

### RNA pull-down assay

5 μg of total RNA was mixed with biotin-labeled probes overnight at 4 °C. 75 μL of Pierce™ Protein A/G Magnetic Beads (#88802, Thermo Fisher Scientific, Waltham, MA, USA) were washed with 300 μL of DEPC to remove impurities. The RNA-RNA mixture was then added to the cleaned beads and mixed at 25 °C for 2 h. A magnetic holder was used to adsorb beads, which were then washed with 500 μL of 75% alcohol for final RNA extraction. Biotin-labeled probes were prepared by Biotech (Shanghai, China) and are listed in Supplementary Table S4.

### Western blot

The detailed steps for harvest, extraction, and denaturation of total protein in GCs and western blot are described in our previous study [15]. GAPDH was used as an internal control. The antibodies used for western blots are listed in Supplementary Table S5.

### mRNA stability assay

GCs were treated with actinomycin D (ActD) (#A113142, Aladdin, Shanghai, China) at a final concentration of 5 ng/μL, 10 h after treatment with miR-187 mimics. Then, cells were collected for RNA isolation and detection of TGFBR2 mRNA levels at 0, 2, 4, 6, 8, and 10 h after treatment with ActD. Similarly, KGN cells were co-treated with pGL3-CMV-MREwt and miR-187 mimics, followed by ActD addition. Firefly luciferase gene mRNA levels were measured using qPCR. Primers used for mRNA stability assays are listed in Supplementary Table S6.

### Apoptosis analysis

GCs were digested with trypsin–EDTA (0.5%) (#25200056, Thermo Fisher Scientific, Waltham, MA) at 37 °C, 48 h after transfection. FACS using an Annexin V-FITC/PI Apoptosis Detection Kit (#A211-01, Vazyme, Nanjing, China) was performed according to the manufacturer’s instructions to evaluate GC apoptosis.

### ChIP

ChIP was performed as previously described [23]. A naïve IgG antibody was used as an internal control, and unprocessed chromatin was used as the input control. The antibodies and primers used are listed in Supplementary Tables S5 and S7, respectively.

### Statistical analyses

All data are shown as means ± S.E.M. from three or more independent experiments. Statistical analyses were performed using GraphPad Prism v8.0 software (San Diego, CA, USA) using a two-tailed Student’s *t*-test (for two groups) or one-way analysis of variance (ANOVA) test (for three or more groups).

## Supplementary information

Additional Figures

Additional Tables

## Data Availability

The datasets used and/or analyzed during the current study are available from the corresponding author upon reasonable request.
