# Supplementary material for: A regulatory network controlling ovarian granulosa cell death
Source: Cell Death Discov. 2023 Feb 20;9:70. doi: 10.1038/s41420-023-01346-9 (PMC9941584; doi:10.1038/s41420-023-01346-9)
Supplement: Supplementary file 1 — Additional Figures [file 41420_2023_1346_MOESM1_ESM.docx]

**Supplementary Figure Legends**

**Fig.S1 Mature sequences and genomic organization of miR-187 in vertebrates.**

(A) miR-187 mature sequences are highly conserved among vertebrates. Asterisks indicate the seed sequence. (B) Genomic organization of miR-187 in vertebrates. The approximate distances between miRNAs and nearby protein-coding genes are given. Chromosome strands are indicated by "+" or "-".

**Fig.S2 The original images of western blot in Figures 2 - 5.**

(A) Western blot related to Figure 2C. (B) Western blot related to Figure 2F. (C) Western blot related to Figure 3A. (D) Western blot related to Figure 3B. (E) Western blot related to Figure 4E. (F) Western blot related to Figure 4F. (G) Western blot related to Figure 5A (H) Western blot related to Figure 5B. Immunoblots of TGFBR2, GAPDH, p-SMAD3, t-SMAD3. The red rectangles indicate the original images of the corresponding experiments involved in this study.

**Fig.S3 miR-187 controls TGFBR2 mRNA stability in sow GCs.**

(A) After transfection of miR-187 mimics or mimics NC into GCs for 10 hours, ActD was added, and then TGFBR2 mRNA levels were detected at 0, 2, 4, 6, 8, and 10 h. (B) After co-transfection of plasmid pGL3-CMV-MREwt and miR-187 mimics or mimics NC into KGN cells for 10 hours, ActD was added, and then firefly luciferase gene mRNA levels were detected at 0, 2, 4, 6, 8, and 10 h.

**Fig.S4 KEGG analysis of potential targets of miR-187.**

An online tool kobas (http://kobas.cbi.pku.cn/) was used for KEGG analysis.

**Fig.S5 Prediction of minimum free energy (MFE) between miR-187 and NORHA**

An online tool RNAhybrid (https://bibiserv.cebitec.uni-bielefeld.de/rnahybrid/) was performed.

**Fig.S6 The original images of western blot in Figures 7.**

(A) Western blot related to Figure 6B. (B) Western blot related to Figure 6G. Immunoblots of TGFBR2, p-SMAD3, t-SMAD3, GAPDH. The red rectangles indicate the original images of the corresponding experiments involved in this study.


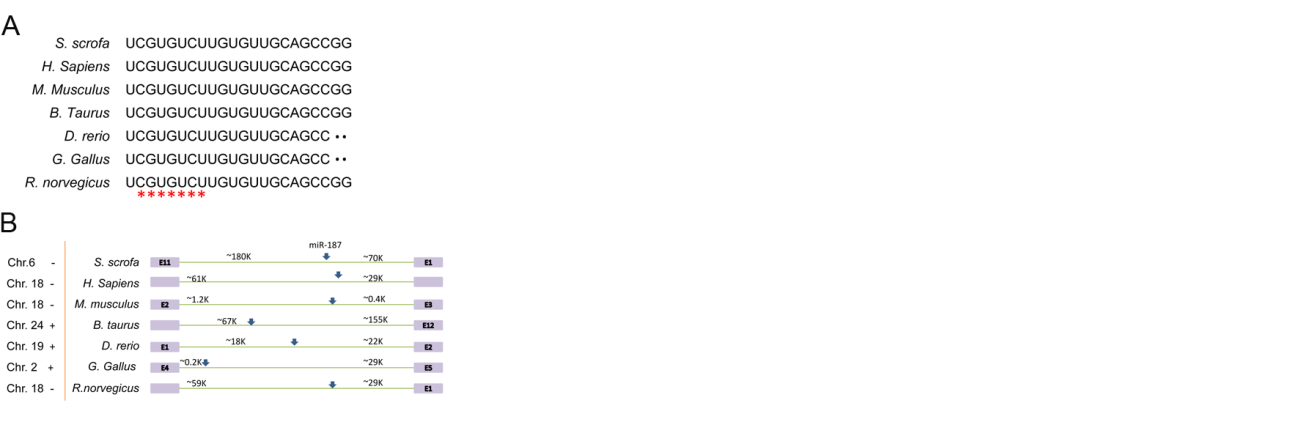


**Fig.S1 Mature sequences and genomic organization of miR-187 in vertebrates.**

**
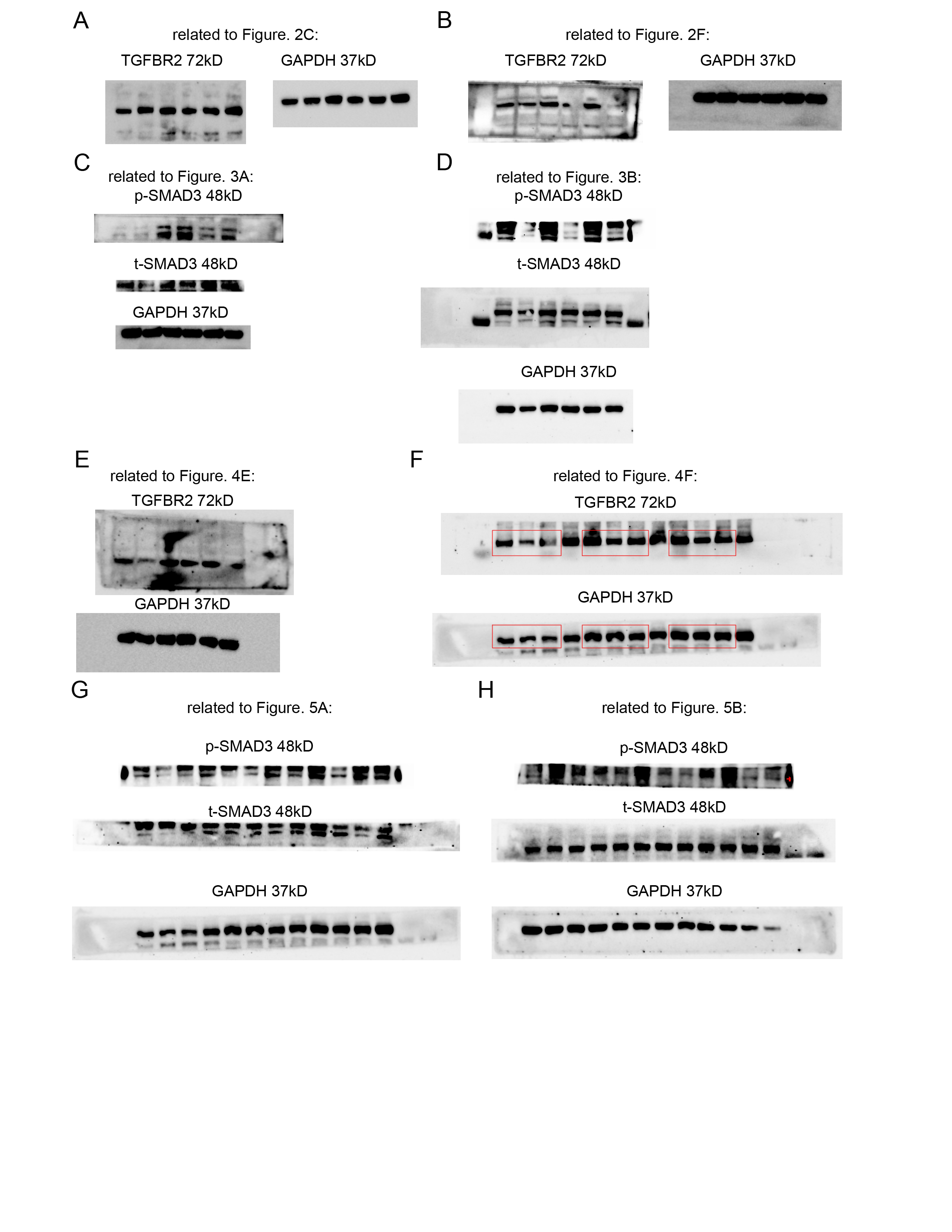
**

**Fig.S2 The original images of western blot in Figures 2 - 5.**


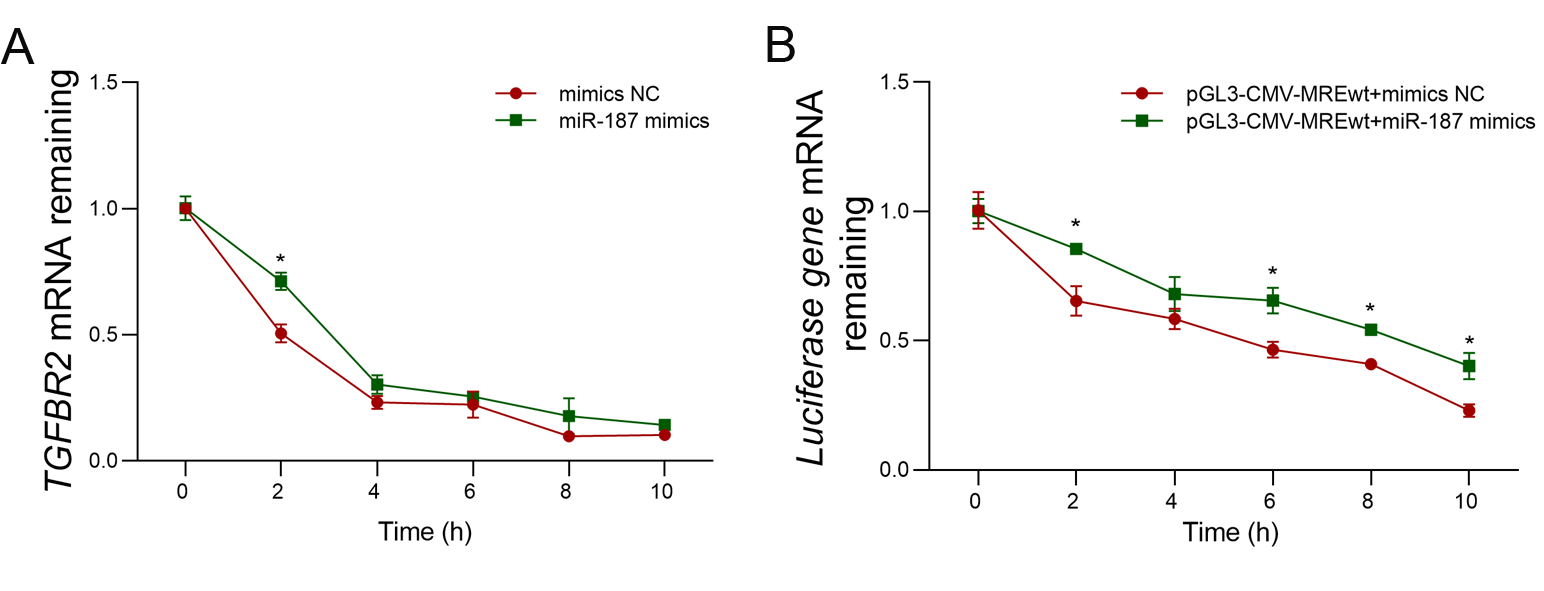


**Fig.S3 miR-187 controls TGFBR2 mRNA stability in sow GCs.**


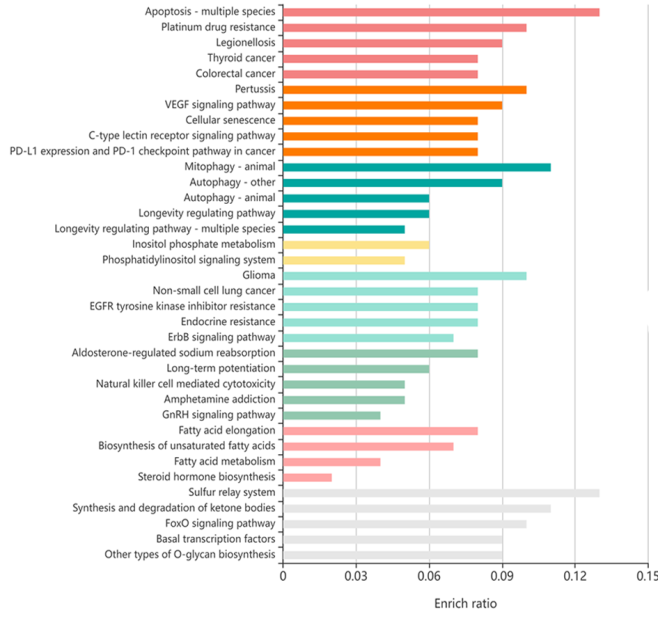


**Fig.S4 KEGG analysis of potential targets of miR-187.**


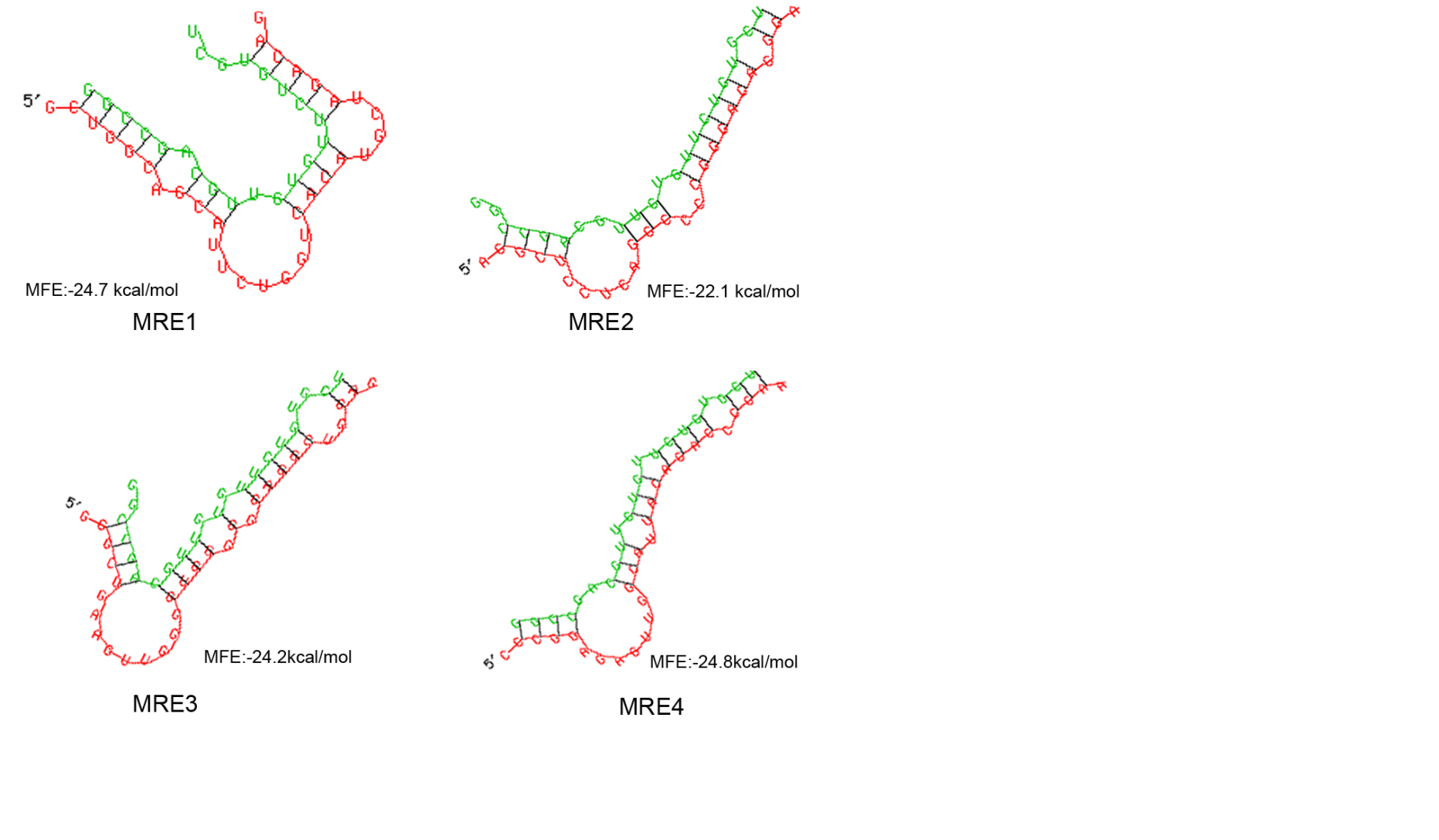


**Fig.S5 Prediction of minimum free energy (MFE) between miR-187 and NORHA**

**
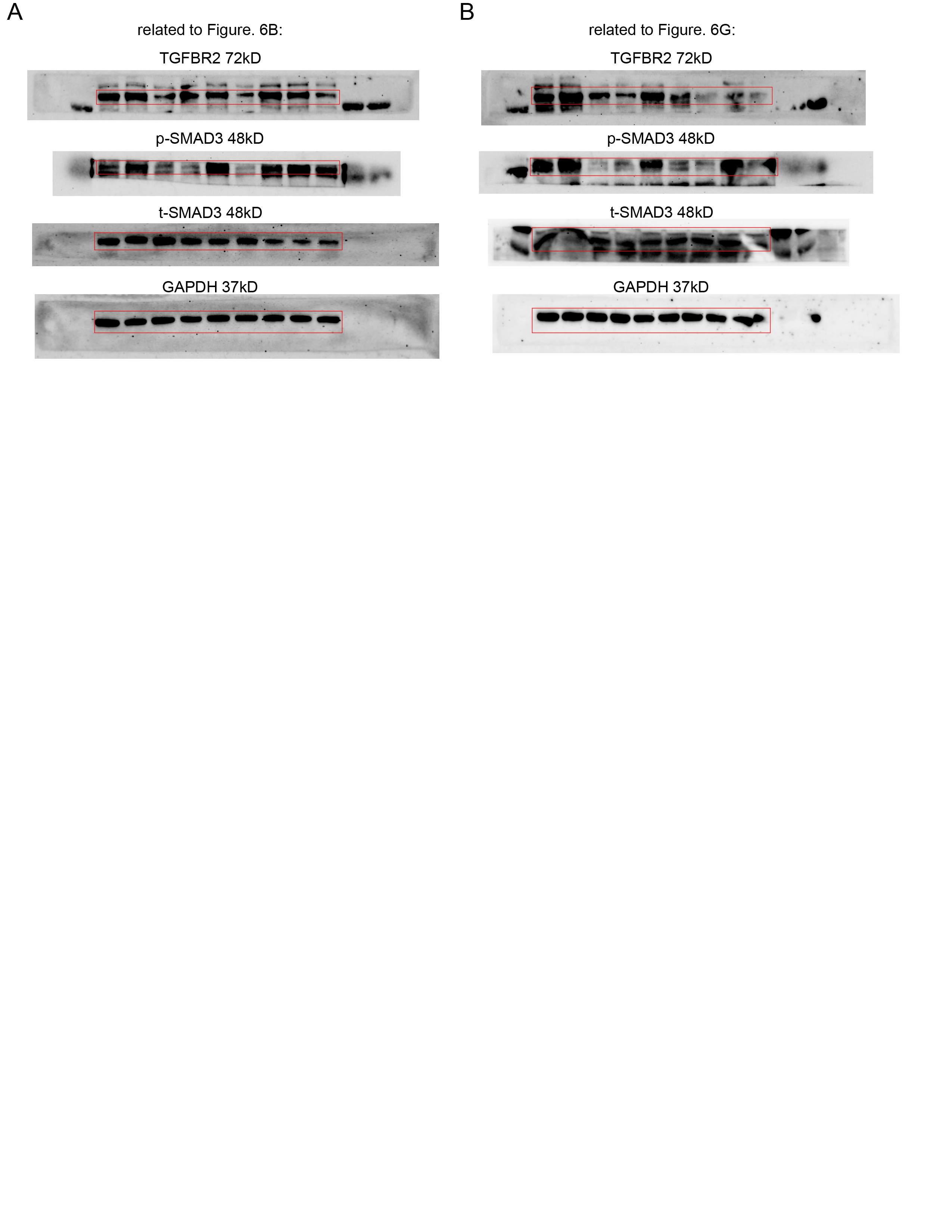
**

**Fig.S6 The original images of western blot in Figures 7.**
