# Supplementary material for: A regulatory network controlling ovarian granulosa cell death
Source: Cell Death Discov. 2023 Feb 20;9:70. doi: 10.1038/s41420-023-01346-9 (PMC9941584; doi:10.1038/s41420-023-01346-9)
Supplement: Supplementary file 2 — Additional Tables [file 41420_2023_1346_MOESM2_ESM.docx]

**Supplementary Table legends**

**Table S1.** Primers used for plasmid construction.

**Table S2.** Oligonucleotide sequences.

**Table S3.** Primers designed for reverse-transcription and qPCR.

**Table S4.** Biotin-labelled probes sequences.

**Table S5.** Antibody Information sheet.

**Table S6.** Primers for mRNA stability assay.

**Table S7.** Primers for ChIP.

**Table S8.** The miRNA response elements (MREs) in TGFBR2 5’-UTR.

**Table S9.** The potential targets of miR-187.

**Table S1**

Primers used for plasmid construction.

| **Primer sequence (5’ to 3’)** | **Tm (℃)** | **Length (bp)** |
| --- | --- | --- |
| F: CTCTATCGATAGGTACCGTTGACATTGATTATTGACTAG | 71 | 620 |
| R: CACGCGTAAGAGCTCGAGCTCTGCTTATATAGACCTCCC |  |  |

Tm, melting temperature; bp, base pair; F, forward primer; R, reverse primer.

**Table S2**

Oligonucleotide sequences.

| **Name** | **Sequence (5’ to 3’)** |
| --- | --- |
| mimics NC | UUCUCCGAACGUGUCACGUTT |
|  | ACGUGACACGUUCGGAGAATT |
| miR-187 mimics | UCGUGUCUUGUGUUGCAGCCGG |
|  | GGCUGCAACACAAGACACGAUU |
| Inhibitor NC | CAGUACUUUUGUGUAGUACAA |
| miR-187 inhibitor | CCGGCUGCAACACAAGACACGA |
| NC siRNA | UUCUCCGAACGUGUCACGUTT |
|  | ACGUGACACGUUCGGAGAATT |
| TGFBR2 siRNA | GGAGGAAGAAUGACGAGAATT |
|  | UGUGGUUGAUGUUGUUGGCTT |
| SMAD4 siRNA | CACCAGGAAUUGAUCUCUCAGGAUU |
|  | AAUCCUGAGAGAUCAAUUCCUGGUG |
| NORHA siRNA | GGAAAUAGCGAGUUGGGUUTT |
|  | AACCCAACUCGCUAUUUCCTT |
| NORFA siRNA | CAGACAGAUGUGGAUGAAUTT |
|  | AUUCAUCCACAUCUGUCUGTT |
| NFIX siRNA | CCAACCGGUUUGUCAGCAUTT |
|  | AUGCUGACAAACCGGUUGGTT |

**Table S3**

Primers designed for reverse-transcription and qPCR.

| **Genes** | **Primer sequence (5’ to 3’)** | **Usage** | **Tm (℃)** |
| --- | --- | --- | --- |
| miR-187 | CCTGTTGTCTCCAGCCACAAAAGAGCACAATATATTTCAGGAGACAACAGGCAGCCGG | RT |  |
| miR-187 | F: CGGGCTCGTGTCTTGTGTTG | qPCR | 60 |
|  | R: CAGCCACAAAAGAGCACAAT |  |  |
| TGFBR2 | F: TGGCTCCTGAAGTCCTAGAGT | qPCR | 60 |
|  | R: GAACCAAAGGGTGGCTCAT |  |  |
| U6 | F: GCTTCGGCAGCACATATACT | qPCR | 60 |
|  | R: TTCACGAATTTGCGTGTCAT |  |  |
| GAPDH | F: CGTGCGGTTGTGGATCT  R: CTCAGTGTAGCCCAGGAT | qPCR | 60 |
| NORHA | F: TCATCCTGCAGCCCTCATTATAC | qPCR | 60 |
|  | R: CCTTCTCAACCCAACTCGCTATT |  |  |
| NORFA | F: TCCGCCCCCAAAATGTGT | qPCR | 60 |
|  | R: CTTGGCACTGCTGGGGCT |  |  |

Tm, melting temperature; RT, reverse transcription; F, forward primer; R, reverse primer.

**Table S4**

Biotin-labelled probes sequences.

| **Targets** | **Primer sequence (5’ to 3’)** |
| --- | --- |
| NORHA | TTTTTTCGGGTCTGTAATGC |
| miR-187 | CCGGCTGCAACACAAGACACGA |

**Table S5**

Antibody Information sheet.

| **Antibodies** | **Company** | **Catalog** | **Dilution** |
| --- | --- | --- | --- |
| anti-TGFBR2 | Santa Cruz | sc-400 | 1:1000 |
| anti-GAPDH | Sangon Biotech | D198662 | 1:3000 |
| anti-SMAD3 | Sangon Biotech | D155234 | 1:1000 |
| anti-p-SMAD3 | Sangon Biotech | D155153 | 1:1000 |
| Goat Anti-Mouse | Yifeixue Biotechnology | YFSA01 | 1:2000 |
| Goat Anti-Rabbit | Yifeixue Biotechnology | YFSA02 | 1:2000 |
| IgG | Santa Cruz | sc-2358 | 1:1000 |

**Table S6**

Primers for mRNA stability assay.

| **Targeted gene** | **Primer sequence (5’ to 3’)** | **Tm (℃)** |
| --- | --- | --- |
| Luciferase gene | F: CAGAGCGAGGTATGTAGGCG | 60 |
|  | R: TTTTCGTTCCACTGAGCGTC |  |
| TGFBR2 | F: TGGCTCCTGAAGTCCTAGAGT  R: GAACCAAAGGGTGGCTCAT | 60 |

Tm, melting temperature; F, forward primer; R, reverse primer.

**Table S7**

Primers for ChIP.

| **Primer sequence (5’ to 3’)** | **Length (bp)** | **Tm (℃)** |
| --- | --- | --- |
| F: GAGCCTATCTGCTGAGCGTTTCT | 298 | 62 |
| R: TGGCCTGCCAACACCTGC |  |  |

bp, base pair; Tm, melting temperature; F, forward primer; R, reverse primer.

**Table S8**

The miRNA response elements (MREs) in TGFBR2 5’-UTR.

| **miRNA** | **Location** | **MFE (kcal/mol)** |
| --- | --- | --- |
| miR-187 | 102-134 | -31.3 |
| miR-8109 | 97-116 | -30.2 |
| miR-6642-3p | 90-110 | -29.6 |
| miR-9639-5p | 119-137 | -28.4 |
| miR-12279-5p | 20-36 | -28.2 |
| miR-5404 | 104-118 | -27.8 |
| miR-1394 | 103-124 | -26.9 |
| miR-718 | 26-45 | -24.2 |
| miR-4066-5p | 115-134 | -23.4 |
| miR-6724-5p | 98-119 | -22.9 |
| miR-12263-5p | 115-134 | -22.7 |
| miR-6366 | 19-36 | -22.2 |
| miR-6729-5p | 131-148 | -21.7 |
| miR-12245-3p | 127-142 | -20.8 |
| miR-H10 | 123-138 | -20.5 |
| miR-3187-3p | 97-116 | -19.5 |
| miR-6127 | 90-110 | -19.1 |
| miR-6642-5p | 119-137 | -14.2 |
| miR-204-2-3p | 20-36 | -12 |
| miR-12244-5p | 104-118 | -11.3 |
| miR-1181 | 103-124 | -10.2 |
| miR-1581 | 26-45 | -9.6 |
| miR-2531 | 115-134 | -8.4 |
| miR-9945 | 98-119 | -8.2 |

MFE, Minimum free energy.

**Table S9**

The potential targets of miR-187.

| **Targets** | **Gene name** |
| --- | --- |
| RTKN2 | rhotekin 2 |
| CCDC169-SOHLH2 | CCDC169-SOHLH2 readthrough |
| CD276 | CD276 molecule |
| SOHLH2 | spermatogenesis and oogenesis specific basic helix-loop-helix 2 |
| CAVIN1 | caveolae associated protein 1 |
| ABHD18 | abhydrolase domain containing 18 |
| ABHD13 | abhydrolase domain containing 13 |
| MEGF10 | multiple EGF like domains 10 |
| LRFN1 | leucine rich repeat and fibronectin type III domain containing 1 |
| LEPR | leptin receptor |
| PDF | peptide deformylase, mitochondrial |
| COG8 | component of oligomeric golgi complex 8 |
| RASD1 | ras related dexamethasone induced 1 |
| NFKBIZ | NFKB inhibitor zeta |
| SLC17A9 | solute carrier family 17 member 9 |
| GAB3 | GRB2 associated binding protein 3 |
| ZNF41 | zinc finger protein 41 |
| CC2D1B | coiled-coil and C2 domain containing 1B |
| TUBGCP3 | tubulin gamma complex associated protein 3 |
| FAM19A3 | family with sequence similarity 19 member A3, C-C motif chemokine like |
| CHAD | chondroadherin |
| RIMKLB | ribosomal modification protein rimK like family member B |
| EML2 | EMAP like 2 |
| DYRK2 | dual specificity tyrosine phosphorylation regulated kinase 2 |
| CHMP2B | charged multivesicular body protein 2B |
| SLC36A2 | solute carrier family 36 member 2 |
| ACTR1A | ARP1 actin related protein 1 homolog A |
| NEUROG2 | neurogenin 2 |
| MAN1B1 | mannosidase alpha class 1B member 1 |
| USP13 | ubiquitin specific peptidase 13 |
| NAV1 | neuron navigator 1 |
| USH2A | usherin |
| ATP2B4 | ATPase plasma membrane Ca2+ transporting 4 |
| ARRDC3 | arrestin domain containing 3 |
| GLRX | glutaredoxin |
| XKRY | XK related, Y-linked |
| C1S | complement C1s |
| ATP11A | ATPase phospholipid transporting 11A |
| XKRY2 | XK related, Y-linked 2 |
| SCARB2 | scavenger receptor class B member 2 |
| KHDRBS3 | KH RNA binding domain containing, signal transduction associated 3 |
| MAPK14 | mitogen-activated protein kinase 14 |
| CDC45 | cell division cycle 45 |
| MINPP1 | multiple inositol-polyphosphate phosphatase 1 |
| SCNM1 | sodium channel modifier 1 |
| COL12A1 | collagen type XII alpha 1 chain |
| HNRNPA2B1 | heterogeneous nuclear ribonucleoprotein A2/B1 |
| RPP25 | ribonuclease P and MRP subunit p25 |
| SPRED1 | sprouty related EVH1 domain containing 1 |
| ASB6 | ankyrin repeat and SOCS box containing 6 |
| FIGN | fidgetin, microtubule severing factor |
| EML1 | EMAP like 1 |
| TOMM34 | translocase of outer mitochondrial membrane 34 |
| CGN | cingulin |
| TMEFF1 | transmembrane protein with EGF like and two follistatin like domains 1 |
| IMPA1 | inositol monophosphatase 1 |
| RAB7A | member RAS oncogene family |
| C14orf139 | spectrin repeat containing nuclear envelope family member 3 |
| CCDC127 | coiled-coil domain containing 127 |
| PRKD3 | protein kinase D3 |
| NR4A1 | nuclear receptor subfamily 4 group A member 1 |
| ATP6AP1 | ATPase H+ transporting accessory protein 1 |
| C1orf57 | nucleoside-triphosphatase, cancer-related |
| MAST2 | microtubule associated serine/threonine kinase 2 |
| MAPRE2 | microtubule associated protein RP/EB family member 2 |
| PHF16 | jade family PHD finger 3 |
| ZNF706 | zinc finger protein 706 |
| TIMELESS | timeless circadian regulator |
| SEMA4C | semaphorin 4C |
| TBC1D22B | TBC1 domain family member 22B |
| GNL3L | G protein nucleolar 3 like |
| MOBKL3 | MOB family member 4, phocein |
| ATG5 | autophagy related 5 |
| AFF4 | AF4/FMR2 family member 4 |
| FBXO21 | F-box protein 21 |
| PLEKHA1 | pleckstrin homology domain containing A1 |
| VOPP1 | VOPP1 WW domain binding protein |
| ETF1 | eukaryotic translation termination factor 1 |
| SEC24C | SEC24 homolog C, COPII coat complex component |
| EFNB2 | ephrin B2 |
| IQGAP1 | IQ motif containing GTPase activating protein 1 |
| NADK | NAD kinase |
| QKI | KH domain containing RNA binding |
| AKAP1 | A-kinase anchoring protein 1 |
| WHSC1 | Wolf-Hirschhorn syndrome candidate 1 |
| HARBI1 | harbinger transposase derived 1 |
| TSC22D1 | TSC22 domain family member 1 |
| PPARG | peroxisome proliferator activated receptor gamma |
| CD2AP | CD2 associated protein |
| RUVBL2 | RuvB like AAA ATPase 2 |
| KIAA1012 | trafficking protein particle complex subunit 8 |
| NEDD4 | NEDD4 E3 ubiquitin protein ligase |
| PRDM1 | PR/SET domain 1 |
| SNORA38 | small nucleolar RNA, H/ACA box 38 |
| POF1B | POF1B actin binding protein |
| DSTYK | dual serine/threonine and tyrosine protein kinase |
| SH3D19 | SH3 domain containing 19 |
| PLEKHH2 | pleckstrin homology, MyTH4 and FERM domain containing H2 |
| CSDE1 | cold shock domain containing E1 |
| C9orf96 | serine/threonine kinase like domain containing 1 |
| PCMTD1 | protein-L-isoaspartate (D-aspartate) O-methyltransferase domain containing 1 |
| GTF2A1 | general transcription factor IIA subunit 1 |
| DCTN4 | dynactin subunit 4 |
| RNF19B | ring finger protein 19B |
| SSTR1 | somatostatin receptor 1 |
| ACVR2B | activin A receptor type 2B |
| EIF2S1 | eukaryotic translation initiation factor 2 subunit alpha |
| SPIB | Spi-B transcription factor |
| NUPR1 | nuclear protein 1, transcriptional regulator |
| BCL2L13 | BCL2 like 13 |
| ZXDA | zinc finger X-linked duplicated A |
| CERK | ceramide kinase |
| JAKMIP3 | Janus kinase and microtubule interacting protein 3 |
| INPP5B | inositol polyphosphate-5-phosphatase B |
| PRR14L | proline rich 14 like |
| ILDR2 | immunoglobulin like domain containing receptor 2 |
| VGLL3 | vestigial like family member 3 |
| MAD2L2 | MAD2 mitotic arrest deficient-like 2 (yeast) |
| STOML2 | stomatin (EPB72)-like 2 |
| TUBG1 | tubulin, gamma 1 |
| FBN2 | fibrillin 2 |
| CD44 | CD44 molecule (Indian blood group) |
| UBLCP1 | ubiquitin like domain containing CTD phosphatase 1 |
| RASGEF1A | RasGEF domain family member 1A |
| FAM127B | retrotransposon Gag like 8A |
| GPATCH2 | G-patch domain containing 2 |
| SH2D4A | SH2 domain containing 4A |
| GALC | galactosylceramidase |
| RICTOR | RPTOR independent companion of MTOR complex 2 |
| DOT1L | DOT1 like histone lysine methyltransferase |
| EIF4A1 | eukaryotic translation initiation factor 4A1 |
| MGRN1 | mahogunin ring finger 1 |
| TNFAIP1 | TNF alpha induced protein 1 |
| ANKRD28 | ankyrin repeat domain 28 |
| USP42 | ubiquitin specific peptidase 42 |
| CITED2 | Cbp/p300 interacting transactivator with Glu/Asp rich carboxy-terminal domain 2 |
| COPS7B | COP9 signalosome subunit 7B |
| CMTM4 | CKLF like MARVEL transmembrane domain containing 4 |
| LPCAT1 | lysophosphatidylcholine acyltransferase 1 |
| KDM6A | lysine demethylase 6A |
| KDM4A | lysine demethylase 4A |
| UBE2V2 | ubiquitin conjugating enzyme E2 V2 |
| SSR3 | signal sequence receptor subunit 3 |
| ZMYND19 | zinc finger MYND-type containing 19 |
| KIAA0146 | scaffold protein involved in DNA repair |
| VPS33A | VPS33A core subunit of CORVET and HOPS complexes |
| DLG5 | discs large MAGUK scaffold protein 5 |
| LOC152217 | NCBP2 antisense 2 |
| LUZP1 | leucine zipper protein 1 |
| POFUT1 | protein O-fucosyltransferase 1 |
| SNORD15B | small nucleolar RNA, C/D box 15B |
| TMPO | thymopoietin |
| ELOVL5 | ELOVL fatty acid elongase 5 |
| UBE2G1 | ubiquitin conjugating enzyme E2 G1 |
| NBL1 | DAN family BMP antagonist |
| RQCD1 | Cell differentiation protein RCD1 |
| ZBTB5 | zinc finger and BTB domain containing 5 |
| FBXW4 | F-box and WD repeat domain containing 4 |
| C4orf41 | trafficking protein particle complex subunit 11 |
| SMC5 | structural maintenance of chromosomes 5 |
| SPRY1 | sprouty homolog 1, antagonist of FGF signaling (Drosophila) |
| APOL6 | apolipoprotein L6 |
| PCNXL2 | pecanex-like 2 (Drosophila) |
| MIB1 | MIB E3 ubiquitin protein ligase 1 |
| TSC22D2 | TSC22 domain family member 2 |
| SPIRE1 | spire type actin nucleation factor 1 |
| SLC39A1 | solute carrier family 39 member 1 |
| CDC42SE2 | CDC42 small effector 2 |
| NOTCH2 | notch receptor 2 |
| RBPJ | recombination signal binding protein for immunoglobulin kappa J region |
| FAM117A | family with sequence similarity 117 member A |
| LOC221710 | small integral membrane protein 13 |
| FAM69B | family with sequence similarity 69 member B |
| CSRNP2 | cysteine and serine rich nuclear protein 2 |
| DFFB | DNA fragmentation factor subunit beta |
| RDX | radixin |
| SGK1 | serum/glucocorticoid regulated kinase 1 |
| BNIP3 | BCL2 interacting protein 3 |
| PAPD4 | poly(A) RNA polymerase D4, non-canonical |
| SFRS2B | serine and arginine rich splicing factor 8 |
| FMNL2 | formin like 2 |
| QSER1 | glutamine and serine rich 1 |
| FAM160A1 | family with sequence similarity 160 member A1 |
| PPP3CA | protein phosphatase 3 catalytic subunit alpha |
| SERBP1 | SERPINE1 mRNA binding protein 1 |
| ZNRF2 | zinc and ring finger 2 |
| GFPT2 | glutamine-fructose-6-phosphate transaminase 2 |
| DAB1 | DAB adaptor protein 1 |
| RAB5B | member RAS oncogene family |
| DCAF13 | DDB1 and CUL4 associated factor 13 |
| GLCE | glucuronic acid epimerase |
| GPRC5B | G protein-coupled receptor class C group 5 member B |
| LIMA1 | LIM domain and actin binding 1 |
| DHX33 | DEAH-box helicase 33 |
| SEC61A1 | SEC61 translocon subunit alpha 1 |
| MGA | MAX dimerization protein MGA |
| CASP8 | caspase 8 |
| MLLT3 | MLLT3 super elongation complex subunit |
| RDH13 | retinol dehydrogenase 13 |
| CHCHD10 | coiled-coil-helix-coiled-coil-helix domain containing 10 |
| CDKN2B | cyclin dependent kinase inhibitor 2B |
| CDK17 | cyclin dependent kinase 17 |
| UCHL1 | ubiquitin C-terminal hydrolase L1 |
| BMP1 | bone morphogenetic protein 1 |
| FRMD6 | FERM domain containing 6 |
| SMAD1 | SMAD family member 1 |
| USP14 | ubiquitin specific peptidase 14 |
| BHLHE40 | basic helix-loop-helix family member e40 |
| ATP6V0E1 | ATPase H+ transporting V0 subunit e1 |
| PRR11 | proline rich 11 |
| CYP4F22 | cytochrome P450 family 4 subfamily F member 22 |
| CCNL1 | cyclin L1 |
| THEM4 | thioesterase superfamily member 4 |
| TMEM129 | transmembrane protein 129, E3 ubiquitin ligase |
| DHX36 | DEAH-box helicase 36 |
| COX4NB | ER membrane protein complex subunit 8 |
| RAB2A | member RAS oncogene family |
| FURIN | paired basic amino acid cleaving enzyme |
| SGMS1 | sphingomyelin synthase 1 |
| WDR67 | TBC1 domain family member 31 |
| EHMT1 | euchromatic histone lysine methyltransferase 1 |
| KTN1 | kinectin 1 |
| NIPBL | Nipped-B homolog (Drosophila) |
| KLF5 | Kruppel like factor 5 |
| CNRIP1 | cannabinoid receptor interacting protein 1 |
| SLC25A26 | solute carrier family 25 member 26 |
| MBD6 | methyl-CpG binding domain protein 6 |
| FGFRL1 | fibroblast growth factor receptor like 1 |
| OXSR1 | oxidative stress responsive kinase 1 |
| KCTD1 | potassium channel tetramerization domain containing 1 |
| SNX25 | sorting nexin 25 |
| PSAP | prosaposin |
| PMAIP1 | phorbol-12-myristate-13-acetate-induced protein 1 |
| RHOH | ras homolog family member H |
| ODC1 | ornithine decarboxylase 1 |
| GPAM | glycerol-3-phosphate acyltransferase, mitochondrial |
| RNF220 | ring finger protein 220 |
| MAPK1 | mitogen-activated protein kinase 1 |
| CMTM6 | CKLF like MARVEL transmembrane domain containing 6 |
| MBNL1 | muscleblind like splicing regulator 1 |
| CEP135 | centrosomal protein 135 |
| CFL2 | cofilin 2 |
| IFNAR1 | interferon alpha and beta receptor subunit 1 |
| EIF2S2 | eukaryotic translation initiation factor 2 subunit beta |
| MSL3 | MSL complex subunit 3 |
| EIF2C1 | argonaute RISC component 1 |
| TSPYL1 | TSPY like 1 |
| PUS7L | pseudouridine synthase 7 like |
| EXOSC2 | exosome component 2 |
| GRSF1 | G-rich RNA sequence binding factor 1 |
| YWHAE | tyrosine 3-monooxygenase/tryptophan 5-monooxygenase activation protein epsilon |
| CNTD1 | cyclin N-terminal domain containing 1 |
| TTL | tubulin tyrosine ligase |
| BOD1 | biorientation of chromosomes in cell division 1 |
| CCDC74A | coiled-coil domain containing 74A |
| DAB2 | DAB adaptor protein 2 |
| EARS2 | glutamyl-tRNA synthetase 2, mitochondrial |
| GAS1 | growth arrest specific 1 |
| GRAMD4 | GRAM domain containing 4 |
| ENC1 | ectodermal-neural cortex 1 |
| FAM134A | family with sequence similarity 134 member A |
| RREB1 | ras responsive element binding protein 1 |
| CNOT6L | CCR4-NOT transcription complex subunit 6 like |
| SLC2A3 | solute carrier family 2 member 3 |
| TMEM65 | transmembrane protein 65 |
| DNAJB2 | DnaJ heat shock protein family (Hsp40) member B2 |
| ETS1 | ETS proto-oncogene 1, transcription factor |
| CPS1 | carbamoyl-phosphate synthase 1 |
| EXOSC10 | exosome component 10 |
| TRIM37 | tripartite motif containing 37 |
| INO80 | INO80 complex ATPase subunit |
| RALGAPB | Ral GTPase activating protein non-catalytic subunit beta |
| SUSD5 | sushi domain containing 5 |
| IDH3B | isocitrate dehydrogenase (NAD(+)) 3 non-catalytic subunit beta |
| TCTEX1D2 | Tctex1 domain containing 2 |
| FNDC3B | fibronectin type III domain containing 3B |
| MTOR | mechanistic target of rapamycin kinase |
| ABCA11P | ATP binding cassette subfamily A member 11 |
| JMJD1C | jumonji domain containing 1C |
| PAQR4 | progestin and adipoQ receptor family member 4 |
| MACF1 | microtubule actin crosslinking factor 1 |
| MARK3 | microtubule affinity regulating kinase 3 |
| UBXN2A | microtubule affinity regulating kinase 3 |
| TRIB2 | tribbles pseudokinase 2 |
| PDE3A | phosphodiesterase 3A |
| CIAO1 | cytosolic iron-sulfur assembly component 1 |
| VPS37B | VPS37B subunit of ESCRT-I |
| ADNP | activity dependent neuroprotector homeobox |
| CCDC117 | coiled-coil domain containing 117 |
| COL3A1 | collagen type III alpha 1 chain |
| CARM1 | coactivator associated arginine methyltransferase 1 |
| BTBD3 | BTB domain containing 3 |
| GOLM1 | golgi membrane protein 1 |
| CRMP1 | collapsin response mediator protein 1 |
| NRG2 | neuregulin 2 |
| BARX2 | BARX homeobox 2 |
| FGF9 | fibroblast growth factor 9 |
| SORCS1 | sortilin-related VPS10 domain containing receptor 1 |
| BCL6 | B-cell CLL/lymphoma 6 |
| THNSL2 | threonine synthase-like 2 (S. cerevisiae) |
| GTDC1 | glycosyltransferase-like domain containing 1 |
| FRMPD3 | FERM and PDZ domain containing 3 |
| AGO1 | argonaute RISC catalytic component 1 |
| NOP9 | NOP9 nucleolar protein |
| ACHE | acetylcholinesterase |
| NOL7 | nucleolar protein 7 |
| FAM116A | DENN domain containing 6A |
| RAD50 | RAD50 double strand break repair protein |
| PSD3 | pleckstrin and Sec7 domain containing 3 |
| RAI1 | retinoic acid induced 1 |
| EPB41 | erythrocyte membrane protein band 4.1 |
| C6orf115 | ABRA C-terminal like |
| HDGF | heparin binding growth factor |
| PRDM13 | PR/SET domain 13 |
| CC2D2A | coiled-coil and C2 domain containing 2A |
| ANKH | ANKH inorganic pyrophosphate transport regulator |
| MTAP | methylthioadenosine phosphorylase |
| LIN9 | lin-9 DREAM MuvB core complex component |
| C5orf22 | chromosome 5 open reading frame 22 |
| TPM1 | tropomyosin 1 |
| SFRS16 | splicing factor, arginine/serine-rich 16 |
| C1orf63 | arginine and serine rich protein 1 |
| SPTY2D1 | SPT2 chromatin protein domain containing 1 |
| MICAL2 | microtubule associated monooxygenase, calponin and LIM domain containing 2 |
| ZBTB8OS | zinc finger and BTB domain containing 8 opposite strand |
| NAA16 | N-alpha-acetyltransferase 16, NatA auxiliary subunit |
| ASCC1 | activating signal cointegrator 1 complex subunit 1 |
| CPSF1 | cleavage and polyadenylation specific factor 1 |
| WDR43 | WD repeat domain 43 |
| FAM91A2 | long intergenic non-protein coding RNA 869 |
| XPO4 | exportin 4 |
| RPS27A | ribosomal protein S27a |
| NHLRC2 | NHL repeat containing 2 |
| RNF103 | ring finger protein 103 |
| HOXA4 | homeobox A4 |
| TMEM14B | transmembrane protein 14B |
| C22orf13 | guanylyl cyclase domain containing 1 |
| WIPF2 | WAS/WASL interacting protein family member 2 |
| TRAPPC3 | trafficking protein particle complex subunit 3 |
| ERC1 | ELKS/RAB6-interacting/CAST family member 1 |
| DHRS4L2 | dehydrogenase/reductase 4 like 2 |
| TMEM203 | transmembrane protein 203 |
| PLOD3 | procollagen-lysine,2-oxoglutarate 5-dioxygenase 3 |
| N4BP2L2 | NEDD4 binding protein 2 like 2 |
| SCN1B | sodium voltage-gated channel beta subunit 1 |
| TXNL1 | thioredoxin like 1 |
| TIAL1 | TIA1 cytotoxic granule associated RNA binding protein like 1 |
| SSR1 | signal sequence receptor subunit 1 |
| MPZL1 | myelin protein zero like 1 |
| FRAT2 | FRAT regulator of WNT signaling pathway 2 |
| AAGAB | alpha and gamma adaptin binding protein |
| MSL2 | MSL complex subunit 2 |
| BAK1 | BCL2 antagonist/killer 1 |
| TSHZ1 | teashirt zinc finger homeobox 1 |
| SETD7 | SET domain containing 7, histone lysine methyltransferase |
| URM1 | SET domain containing 7, histone lysine methyltransferase |
| OXCT1 | 3-oxoacid CoA-transferase 1 |
| RHOT1 | ras homolog family member T1 |
| CHEK1 | checkpoint kinase 1 |
| ARID1A | AT-rich interaction domain 1A |
| ZFHX4 | zinc finger homeobox 4 |
| ZFAND5 | zinc finger AN1-type containing 5 |
| EIF5A2 | eukaryotic translation initiation factor 5A2 |
| FANCB | FA complementation group B |
| CAPZB | capping actin protein of muscle Z-line subunit beta |
| SS18L1 | subunit of BAF chromatin remodeling complex |
| ARHGDIA | Rho GDP dissociation inhibitor alpha |
| GABARAPL1 | GABA type A receptor associated protein like 1 |
| DDX55 | DEAD-box helicase 55 |
| EIF3D | eukaryotic translation initiation factor 3 subunit D |
| ABHD14A | abhydrolase domain containing 14A |
| AKT2 | AKT serine/threonine kinase 2 |
| SFXN1 | sideroflexin 1 |
| H2AFV | H2A histone family, member V |
| SEMA3C | semaphorin 3C |
| CCT3 | chaperonin containing TCP1 subunit 3 |
| KIAA0284 | centrosomal protein 170B |
| PCNP | PEST proteolytic signal containing nuclear protein |
| CDH2 | cadherin 2 |
| IFT80 | intraflagellar transport 80 |
| WDFY2 | WD repeat and FYVE domain containing 2 |
| PAN3 | poly(A) specific ribonuclease subunit PAN3 |
| TACC2 | transforming acidic coiled-coil containing protein 2 |
| ZNF684 | zinc finger protein 684 |
| SPHAR | S-phase response |
| MEF2D | myocyte enhancer factor 2D |
| ISL2 | ISL LIM homeobox 2 |
| RAB18 | member RAS oncogene family |
| RPS9 | ribosomal protein S9 |
| RBPMS2 | RNA binding protein, mRNA processing factor 2 |
| C1orf216 | chromosome 1 open reading frame 216 |
| TJP1 | tight junction protein 1 |
| TP53INP1 | tumor protein p53 inducible nuclear protein 1 |
| CCDC74B | coiled-coil domain containing 74B |
| PRPF4B | pre-mRNA processing factor 4B |
| DHX15 | DEAH-box helicase 15 |
| SIN3A | SIN3 transcription regulator family member A |
| HIPK3 | homeodomain interacting protein kinase 3 |
| NOC2L | NOC2 like nucleolar associated transcriptional repressor |
| CD9 | CD9 molecule |
| PPM1E | protein phosphatase, Mg2+/Mn2+ dependent 1E |
| ECHDC1 | ethylmalonyl-CoA decarboxylase 1 |
| CRIM1 | cysteine rich transmembrane BMP regulator 1 |
| ZNF92 | zinc finger protein 92 |
| CAND1 | cullin associated and neddylation dissociated 1 |
| PEX12 | peroxisomal biogenesis factor 12 |
| C4orf49 | mitochondria localized glutamic acid rich protein |
| DPY19L2P2 | DPY19L2 pseudogene 2 |
| PRKCA | protein kinase C alpha |
| EXOSC8 | exosome component 8 |
| TESK2 | testis associated actin remodelling kinase 2 |
| ROBO1 | roundabout guidance receptor 1 |
| SOCS2 | suppressor of cytokine signaling 2 |
| UQCRFS1 | ubiquinol-cytochrome c reductase, Rieske iron-sulfur polypeptide 1 |
| SLCO5A1 | solute carrier organic anion transporter family member 5A1 |
| ABCB7 | ATP binding cassette subfamily B member 7 |
| MNAT1 | MNAT1 component of CDK activating kinase |
| MTHFD1L | methylenetetrahydrofolate dehydrogenase (NADP+ dependent) 1 like |
| CALM1 | calmodulin 1 |
| C12orf23 | transmembrane protein 263 |
| SPRY2 | sprouty RTK signaling antagonist 2 |
| NTN4 | netrin 4 |
| KLHL2 | kelch like family member 2 |
| AKAP2 | A-kinase anchoring protein 2 |
| FGD4 | FYVE, RhoGEF and PH domain containing 4 |
| NUDC | nuclear distribution C, dynein complex regulator |
| PLK3 | polo like kinase 3 |
| NUDT19 | nudix hydrolase 19 |
| ADAM9 | ADAM metallopeptidase domain 9 |
| CREG1 | cellular repressor of E1A stimulated genes 1 |
| ARL2BP | ADP ribosylation factor like GTPase 2 binding protein |
| VRK1 | VRK serine/threonine kinase 1 |
| RPL27A | ribosomal protein L27a |
| STAU2 | staufen double-stranded RNA binding protein 2 |
| PTPN12 | protein tyrosine phosphatase non-receptor type 12 |
| HTRA1 | HtrA serine peptidase 1 |
| GUF1 | GTP binding elongation factor GUF1 |
| HNRNPR | heterogeneous nuclear ribonucleoprotein R |
| SF3A3 | splicing factor 3a subunit 3 |
| PAX9 | paired box 9 |
| CLOCK | clock circadian regulator |
| BNIP2 | BCL2 interacting protein 2 |
| PDSS2 | decaprenyl diphosphate synthase subunit 2 |
| POLR3H | RNA polymerase III subunit H |
| CHD2 | chromodomain helicase DNA binding protein 2 |
| PIGG | phosphatidylinositol glycan anchor biosynthesis class G |
| APC | APC regulator of WNT signaling pathway |
| KLHL29 | kelch like family member 29 |
| SPRYD3 | SPRY domain containing 3 |
| FAM168A | family with sequence similarity 168 member A |
| C16orf13 | methyltransferase like 26 |
| BTC | betacellulin |
| SSH2 | slingshot protein phosphatase 2 |
| ITPKB | inositol-trisphosphate 3-kinase B |
| STAT3 | signal transducer and activator of transcription 3 |
| ZNF711 | zinc finger protein 711 |
| ALMS1 | ALMS1 centrosome and basal body associated protein |
| TIPARP | TCDD inducible poly(ADP-ribose) polymerase |
| HMBOX1 | homeobox containing 1 |
| POLD4 | DNA polymerase delta 4, accessory subunit |
| MDM2 | MDM2 proto-oncogene |
| SEPHS1 | selenophosphate synthetase 1 |
| SLC4A1AP | solute carrier family 4 member 1 adaptor protein |
| PPP2R5A | protein phosphatase 2 regulatory subunit B'alpha |
| SACS | sacsin molecular chaperone |
| FAM174B | family with sequence similarity 174 member B |
| ZNF649 | zinc finger protein 649 |
| GEMIN5 | gem nuclear organelle associated protein 5 |
| RNF169 | ring finger protein 169 |
| EDNRB | endothelin receptor type B |
| ZNF839 | zinc finger protein 839 |
| ARV1 | ARV1 homolog, fatty acid homeostasis modulator |
| FLRT2 | fibronectin leucine rich transmembrane protein 2 |
| HNRNPF | heterogeneous nuclear ribonucleoprotein F |
| MYD88 | MYD88 innate immune signal transduction adaptor |
| PPM1F | protein phosphatase, Mg2+/Mn2+ dependent 1F |
| PPIL2 | peptidylprolyl isomerase like 2 |
| GTF2B | general transcription factor IIB |
| MYH10 | myosin heavy chain 10 |
| CCNDBP1 | cyclin D1 binding protein 1 |
| BACH1 | BTB domain and CNC homolog 1 |
| EEF2 | eukaryotic translation elongation factor 2 |
| MCM2 | minichromosome maintenance complex component 2 |
| ENPP5 | ectonucleotide pyrophosphatase/phosphodiesterase family member 5 |
| HOMER1 | homer scaffold protein 1 |
| ITPRIP | inositol 1,4,5-trisphosphate receptor interacting protein |
| STAU1 | staufen double-stranded RNA binding protein 1 |
| ASXL1 | ASXL transcriptional regulator 1 |
| C19orf50 | KxDL motif containing 1 |
| DYRK1A | dual specificity tyrosine phosphorylation regulated kinase 1A |
| CASP3 | caspase 3 |
| AARS2 | alanyl-tRNA synthetase 2, mitochondrial |
| PHF13 | PHD finger protein 13 |
| GATAD1 | GATA zinc finger domain containing 1 |
| METTL2A | methyltransferase 2A, methylcytidine |
| R3HDM2 | R3H domain containing 2 |
| DUS1L | dihydrouridine synthase 1 like |
| KLC1 | kinesin light chain 1 |
| SLC25A20 | SLC25A20 solute carrier family 25 member 20 |
| NHSL1 | NHS like 1 |
| TBPL1 | TATA-box binding protein like 1 |
| PAFAH1B1 | platelet activating factor acetylhydrolase 1b regulatory subunit 1 |
| ATP2C1 | ATPase secretory pathway Ca2+ transporting 1 |
| HSD17B12 | hydroxysteroid 17-beta dehydrogenase 12 |
| UMPS | uridine monophosphate synthetase |
| SFXN3 | sideroflexin 3 |
| TSR1 | TSR1 ribosome maturation factor |
| PHRF1 | PHD and ring finger domains 1 |
| ABHD6 | abhydrolase domain containing 6, acylglycerol lipase |
| TRIM41 | tripartite motif containing 41 |
| SLC2A1 | solute carrier family 2 member 1 |
| ANK3 | ankyrin 3 |
| GGA2 | golgi associated, gamma adaptin ear containing, ARF binding protein 2 |
| RPRD1A | regulation of nuclear pre-mRNA domain containing 1A |
| PRDM2 | PR/SET domain 2 |
| CNNM4 | cyclin and CBS domain divalent metal cation transport mediator 4 |
| S100A4 | S100 calcium binding protein A4 |
| EXOC7 | exocyst complex component 7 |
| FBXW7 | F-box and WD repeat domain containing 7 |
| FAM98A | family with sequence similarity 98 member A |
| TGFBR2 | transforming growth factor beta receptor 2 |
| GPRIN2 | G protein regulated inducer of neurite outgrowth 2 |
| FAM212B | inka box actin regulator 2 |
| NFAM1 | NFAT activating protein with ITAM motif 1 |
| AQP1 | aquaporin 1 (Colton blood group) |
| FBXO44 | F-box protein 44 |
| STC2 | stanniocalcin 2 |
| PHOSPHO1 | phosphoethanolamine/phosphocholine phosphatase 1 |
| DMBX1 | diencephalon/mesencephalon homeobox 1 |
| DYNLL2 | dynein light chain LC8-type 2 |
| AVPI1 | arginine vasopressin induced 1 |
| BCL2L2-PABPN1 | BCL2L2-PABPN1 readthrough |
| AKIRIN1 | akirin 1 |
| BAZ2A | bromodomain adjacent to zinc finger domain 2A |
| LARP4B | La ribonucleoprotein 4B |
| NRF1 | nuclear respiratory factor 1 |
| PABPN1 | poly(A) binding protein nuclear 1 |
| RPS9 | ribosomal protein S9 |
| CYP4F11 | cytochrome P450 family 4 subfamily F member 11 |
| ELAVL3 | ELAV like RNA binding protein 3 |
| GPATCH2L | G-patch domain containing 2 like |
| GOLGA7B | golgin A7 family member B |
| CTH | cystathionine gamma-lyase |
| ZBTB4 | zinc finger and BTB domain containing 4 |
| ARL10 | ADP ribosylation factor like GTPase 10 |
| SMC1A | structural maintenance of chromosomes 1A |
| AGO1 | argonaute RISC catalytic component 1 |
| CHD8 | chromodomain helicase DNA binding protein 8 |
| OTUD7B | OTU deubiquitinase 7B |
| NAIF1 | nuclear apoptosis inducing factor 1 |
| RCAN1 | regulator of calcineurin 1 |
| SLC39A9 | solute carrier family 39 member 9 |
| LDB2 | LIM domain binding 2 |
| SLC6A4 | solute carrier family 6 member 4 |
| LRRC59 | leucine rich repeat containing 59 |
| FAM57B | TLC domain containing 3B |
| MIF4GD | MIF4G domain containing |
| CBX6 | chromobox 6 |
| ZDHHC3 | zinc finger DHHC-type palmitoyltransferase 3 |
